# Vosoritide treatment for children with hypochondroplasia: a phase 2 trial

**DOI:** 10.1016/j.eclinm.2024.102591

**Published:** 2024-04-11

**Authors:** Andrew Dauber, Anqing Zhang, Roopa Kanakatti Shankar, Kimberly Boucher, Tara McCarthy, Niusha Shafaei, Raheem Seaforth, Meryll Grace Castro, Niti Dham, Nadia Merchant

**Affiliations:** a Division of Endocrinology, Children's National Hospital, Washington, DC 20010, USA; b Department of Pediatrics, The George Washington University School of Medicine and Health Sciences, Washington, DC 20052, USA; c Division of Biostatistics, Children's National Hospital, Washington, DC 20010, USA; d Division of Cardiology, Children's National Hospital, Washington, DC 20010, USA

**Keywords:** Hypochondroplasia, Vosoritide, CNP, C-type natriuretic peptide, FGFR3

## Abstract

**Background:**

Hypochondroplasia is a rare autosomal dominant skeletal dysplasia due to activating variants in *FGFR3*. It presents with disproportionate short stature with a wide range of clinical severity. There are currently no approved medications to treat short stature in children with hypochondroplasia. Vosoritide is a C-type natriuretic peptide analog that was recently approved for improving growth in children with achondroplasia. We aimed to evaluate the safety and efficacy of vosoritide in children with hypochondroplasia.

**Methods:**

We conducted a single-arm, phase 2, open-label trial at a single centre in the USA and enrolled 26 children with hypochondroplasia. The trial consists of a 6-month observation period to establish a baseline annualized growth velocity followed by a 12-month intervention period during which vosoritide is administered daily via subcutaneous injection at a dose of 15 μg/kg/day. The trial's co-primary endpoints included the incidence of adverse events and the change from baseline in age-sex standardized annualized growth velocity and height standardized deviation score (SDS) after 12 months of treatment. This trial is registered with ClinicalTrials.gov (NCT04219007).

**Findings:**

Twenty-four participants with a mean age of 5.86 years received vosoritide therapy. The first participant was enrolled on August 4, 2020, and the final participant completed the 18-month trial on September 8, 2023. Vosoritide was well tolerated with no treatment-related serious adverse events. Injection site reactions occurred in 83.3% of participants. No participants discontinued therapy due to an adverse event. Annualized growth velocity increased by 2.26 standard deviations (SD) and height SDS increased by 0.36 SD during the treatment period versus the observation period. Hypochondroplasia specific height SDS increased by 0.38 SD. There was a 1.81 cm/year increase in absolute annualized growth velocity.

**Interpretation:**

Vosoritide was safe and effective in increasing growth velocity in children with hypochondroplasia. Efficacy was similar to what has been reported in children with achondroplasia.

**Funding:**

This study was supported by an investigator-initiated grant from 10.13039/100008484BioMarin Pharmaceutical.

**Research in context**

**Evidence before this study**

There is a paucity of literature available about the prevalence and natural history of hypochondroplasia. We searched PubMed from the database inception to January 2, 2024 for papers published in English using the terms “hypochondroplasia and prevalence” or “hypochondroplasia and treatment” or “hypochondroplasia and growth hormone”. Our search yielded 51 results. We performed a second search using the term “vosoritide” which yielded 40 results. The available literature suggests that hypochondroplasia is a rare disorder with prevalence estimates ranging from 1:15,000 to 1:100,000 with a wide clinical spectrum. There have been no controlled trials of any medications for hypochondroplasia. Limited data are available about the efficacy of growth hormone in children with hypochondroplasia. Studies were reviewed about the efficacy of vosoritide, a c-type natriuretic peptide (CNP) analog, for the treatment of achondroplasia. No prior studies of vosoritide in hypochondroplasia exist.

**Added value of this study**

This is the first study of vosoritide or any precision medication therapy for hypochondroplasia. This study provides preliminary evidence of the efficacy of vosoritide for improving growth in children with hypochondroplasia. It also confirms the relatively benign short-term safety profile of vosoritide.

**Implications of all the available evidence**

This study supports the development of vosoritide and potentially other CNP based therapies for children with hypochondroplasia.

## Introduction

Hypochondroplasia is a rare, autosomal dominant skeletal dysplasia manifesting with disproportionate short stature, rhizomelic or mesomelic limb shortening, relative macrocephaly, and occasional mild orthopedic manifestations such as tibial bowing and limited elbow extension. The exact prevalence of hypochondroplasia is unknown as there is a wide clinical spectrum and the condition is often underdiagnosed. Prevalence estimates range from 0.2 to 1.3 per 100,000 in birth cohorts up to estimates of 1 in 15,000–40,000 in later childhood, similar to the prevalence of achondroplasia.^1^, ^2^, ^3^ Adult height is significantly impaired with one study estimating a mean adult height of 130.8 cm for females and 143.6 cm for males.^4^ The majority of children with hypochondroplasia harbor activating variants in the *FGFR3* gene, although patients may be diagnosed on clinical and radiological grounds alone.^5^^,^^6^ Activation of FGFR3 and its downstream pathways leads to impaired endochondral ossification resulting in short stature. Individuals with hypochondroplasia have different genetic variants in *FGFR3* than those seen in individuals with achondroplasia, although their clinical spectrums can overlap, with hypochondroplasia generally being less severe. Individuals with achondroplasia may have significant medical comorbidities such as foramen magnum stenosis, sleep apnea, spinal stenosis, genu varum and recurrent otitis media. These are much less common in hypochondroplasia in which disproportionate short stature is the primary clinical concern. To date, there are no approved medications for the treatment of short stature in individuals with hypochondroplasia.

Vosoritide is a c-type natriuretic peptide (CNP) analog that upon binding its receptor on chondrocytes inhibits the mitogen-activated protein kinase (MAPK) pathway. This pathway is downstream of FGFR3 and is activated in hypochondroplasia and achondroplasia. In a Phase 3, placebo-controlled trial, vosoritide was shown to be efficacious in increasing growth velocity in children with achondroplasia with an adjusted mean difference in annualized growth velocity of 1.57 cm/year.^7^ Based on this study and additional trials, vosoritide has now been approved in numerous countries for use in children with achondroplasia. Based on its mechanism of action, vosoritide may be beneficial in any growth disorder with increased MAPK signaling. The current Phase 2 trial was designed to assess the safety and efficacy of vosoritide in children with selected genetic causes of short stature including hypochondroplasia.

## Methods

### Study design

This Phase 2, open label trial is part of a basket trial entitled “Vosoritide for Selected Genetic Causes of Short Stature” (Study Pro00013585, ClinicalTrials.gov number NCT04219007) which is being conducted at a single site, Children's National Hospital, in Washington, DC. The basket trial includes participants with 6 genetic categories of short stature including: 1. Hypochondroplasia, 2. RASopathies, 3. *ACAN* mutation carriers, 4. Carriers of heterozygous mutations in the *NPR2* gene (the gene that encodes the receptor for CNP), 5. SHOX deficiency, and 6. Carriers of mutations in the *NPPC* gene (the gene that encodes CNP). Study enrollment for all cohorts began in August 2020. Due to the preponderance of participants with hypochondroplasia who were recruited into the study, enrollment of the hypochondroplasia cohort was closed in March 2022 after 26 participants with hypochondroplasia were enrolled. This was done to ensure adequate space in the trial for participants in the other cohorts and was not based on a specific power calculation in this exploratory study. Enrollment in the other cohorts is ongoing. The final participant with hypochondroplasia completed the 18-month study protocol in September 2023. The current manuscript focuses solely on the hypochondroplasia cohort.

The study consists of a 6-month observation period to establish a baseline annualized growth velocity followed by a 12-month intervention period during which vosoritide is administered daily via subcutaneous injection at a dose of 15 μg/kg/day, the same dose as used in the Phase 3 trial of children with achondroplasia.^7^ Participants were seen for study visits at 6-month intervals with frequent, scheduled telephone contact in-between visits to assess treatment adherence and to screen for adverse events. Details of the protocol can be found in the Supplementary Materials.

The study received ethical approval from the Institutional Review Board of Children's National Hospital. Written informed consent was obtained from each participant's parent or legal guardian. Verbal assent was obtained for all children aged 7 years or older, and written assent was obtained once children turned 12 years old.

### Participants

Participants were eligible for inclusion in the study if they met the following major inclusion criteria (additional details in the Supplementary Materials): 1. Age ≥3 years 0 days and <11 years for males and <10 years for females, 2. Pre-pubertal, 3. Standing height ≤−2.25 standard deviation scores (SDS) on the United States CDC growth charts,^8^ 4. Presence of a confirmed mutation in the *FGFR3* gene associated with hypochondroplasia, 5. Absence of growth hormone deficiency. Participants were excluded if they had additional medical conditions which may affect growth, malnutrition defined as a BMI <5th percentile, or a prior history of malignancy. Participants were not allowed to take concomitant growth hormone therapy during the trial, but prior use of growth hormone was permitted.

### Study objectives and outcomes

The study has 3 prespecified co-primary outcomes: 1. To evaluate the safety and tolerability of daily subcutaneous injections of vosoritide administered for 12 months in patients with selected genetic causes of short stature, 2. To evaluate the change from baseline in age-sex standardized annualized growth velocity after 12 months of daily SC injections of vosoritide in patients with selected genetic causes of short stature, and 3. To evaluate the change from baseline in age-sex standardized height SDS after 12 months of daily SC injections of vosoritide in patients with selected genetic causes of short stature. Annualized growth velocity was standardized using data from the First Zurich Longitudinal Study of Growth and Development^9^ as it includes growth velocity data starting at age 2.5 years. Height SDS was calculated using the United States CDC growth charts.^8^ Hypochondroplasia specific height SDS was calculated using recently published hypochondroplasia growth curves.^10^ To measure these outcomes, the following 3 co-primary endpoints were specified: 1. The incidence of adverse events per participant, 2. The change from baseline in age-sex standardized annualized growth velocity after 12 months of treatment, and 3. The change from baseline in age-sex standardized height SDS after 12 months of treatment. Height was measured five times at each study visit on a wall-mounted calibrated Harpenden stadiometer. The average of all five values was used. Safety assessments included routine laboratory evaluations at each visit, baseline and Month 12 echocardiograms, electrocardiograms at each visit, vital signs measurements, and spine X-rays at Day 1 and Month 12.

Secondary outcomes included the change in body proportions as measured by sitting height to standing height ratio and arm span minus standing height, as well as the change in bone age to chronological age ratio after 12 months of therapy. Sitting height was measured three times at each visit using a sitting height table. The average of the three values was used. Sitting height to standing height ratio and arm span minus standing height were standardized for age and sex using available reference data and SDS were calculated.^11^^,^^12^ Bone age X-rays were read by a single reviewer who was blinded to participant ID, age and time point. Exploratory endpoints included pharmacokinetic parameters, pharmacodynamic markers including collagen X biomarker (CXM) levels and urine cGMP production, and changes in quality of life as measured by the QoLISSY scale.^13^ CXM levels in serum were measured in duplicate via an ELISA assay at the Shriners Hospitals for Children in Portland, Oregon as previously described.^14^ The average of the two values was used for analysis. Urine samples were obtained for cGMP measurement at baseline and 60, 120, and 240 min after vosoritide injections at the Day 1, Month 6 and Month 12 visits. cGMP levels were assayed at ICON Laboratories (Farmingdale, NY) and were normalized to urine creatinine levels at each time point.

### Statistical analysis

No formal power calculations were done for this proof of principle study. Sample size was determined based on logistical feasibility. Medication adherence was calculated using the number of returned empty vials divided by the number of days of prescribed therapy. Demographics and clinical characteristics were summarized with mean and standard deviation (SD), median and interquartile (IQR) for numerical variables, frequency and proportion for categorical variables. Primary and secondary clinical outcomes were summarized with mean (SD) for the 6-month observation period and the 12-month treatment period. Paired t-tests were carried out to compare the difference between the treatment and observation periods. The corresponding p-value and 95% confidence interval (CI) were reported. Box and whisker plots were used for both primary endpoints (annualized growth velocity and height SDS) and exploratory endpoints (CXM and cGMP) to visualize the trends for the change from baseline to each of the follow-up visits. Safety data (AEs) were summarized as count and percentage of patients as well as the number of total occurrences per each listed AE for the observation period and the treatment period, respectively. As exploratory analyses, the primary endpoints were stratified based on age, baseline growth velocity and baseline height SDS. The summaries of mean (SD) for each visit, mean differences between treatment and observation periods, and the corresponding p-value and 95% CI were all reported. Parent reported quality of life using the QoLISSY scale was also reported with a similar tabular format. Data were analyzed using SAS for Windows version 9.4 (SAS Institute Inc., Cary, NC). A two-sided test with a significance level of 0.05 was used for all hypothesis tests.

### Role of the funding source

This is an investigator-initiated study funded by a grant from BioMarin Pharmaceutical. The investigator team designed and wrote the protocol, conducted the study, performed all analyses and composed the manuscript. BioMarin played no role in any of these functions. An independent data and safety monitoring board provided oversight and reviewed all data every 6 months.

## Results

### Participants

**Fig. 1:**
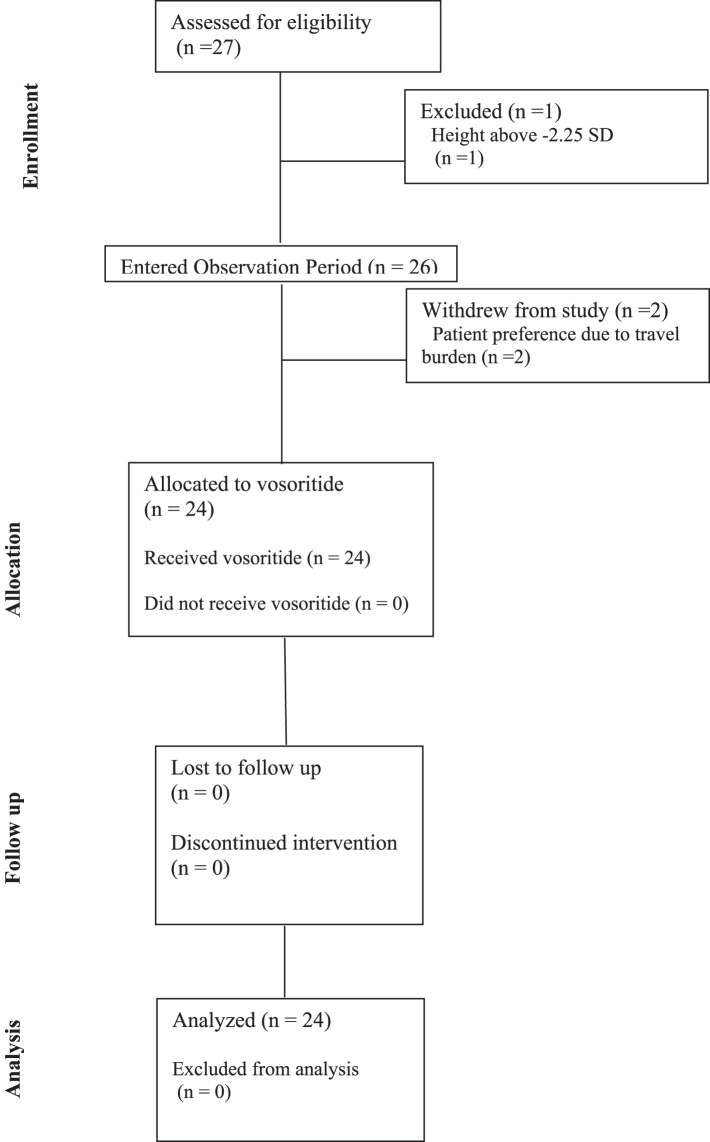
CONSORT diagram.

Twenty-six participants enrolled in the study. The first participant was enrolled on August 4, 2020, and the final participant completed the 18-month trial on September 8, 2023. Two participants withdrew during the observation period due to the travel requirements for the study. Twenty-four participants were initiated on vosoritide, and all 24 participants completed the trial (Fig. 1). All data analyses are based on these 24 participants. Mean (SD) age at screening was 5.9 ± 2.3 years (Table 1). Fifty percent of participants were female, and 22 of the 24 participants had the p.Asn540Lys variant in FGFR3. Mean baseline height was −3.29 ± 0.68 SD (range −4.78 to −2.27 SD). Of the 24 treated participants, 2 had a history of seizures, 3 had ADHD (1 with intellectual disability and dysgenesis of the corpus callosum), and 3 had prior neurosurgical procedures (2 foramen magnum decompressions and 1 posterior fossa fenestration for hydrocephalus). Adherence to daily vosoritide administration was excellent with a mean administration rate of 98.7%. All participants received greater than 98% of prescribed doses except for one participant in whom vosoritide administration was suspended temporarily due to an adverse event (described below).

**Table 1:** Participant demographics and clinical characteristics.

**Total enrolled subjects**	N = 24
**Age at screening (years)**	
Mean (SD); median (IQR)	5.86 (2.29); 5.55 (2.39)
**Age group** # (%)	
3–<5 year	10 (41.7%)
5–<9 year	11 (45.8%)
9–<11 year	3 (12.5%)
**Sex**	
Female	12 (50%)
Male	12 (50%)
**Race**	
Caucasian	17 (70.8%)
Asian	4 (16.7%)
Other	3 (12.5%)
**Ethnicity**	
Non-Hispanic/Latino	23 (95.8%)
Hispanic/Latino	1 (4.2%)
**Previously treated with growth hormone**	
Yes	3 (12.5%)
No	21 (87.5%)
**Genetic Variant**	
Asn540Lys	22 (91.7%)
Gly342Cys	1 (4.2%)
Ser351Phe	1 (4.2%)

### Height efficacy outcomes

**Fig. 2:**
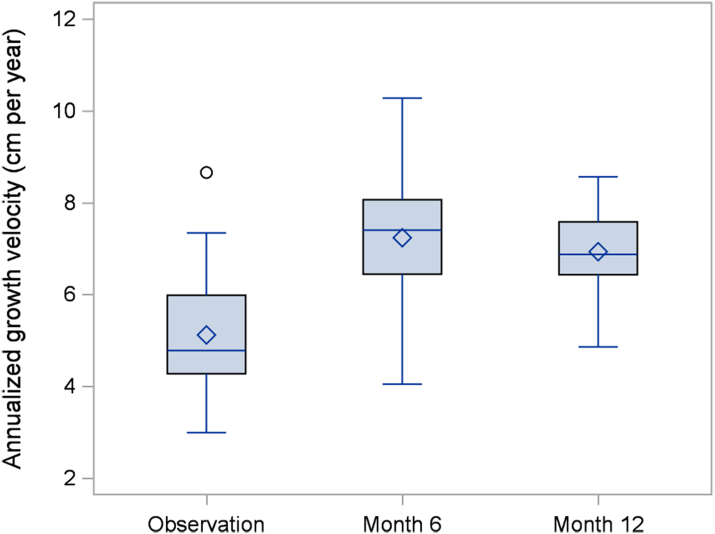
Annualized growth velocity.

**Fig. 3:**
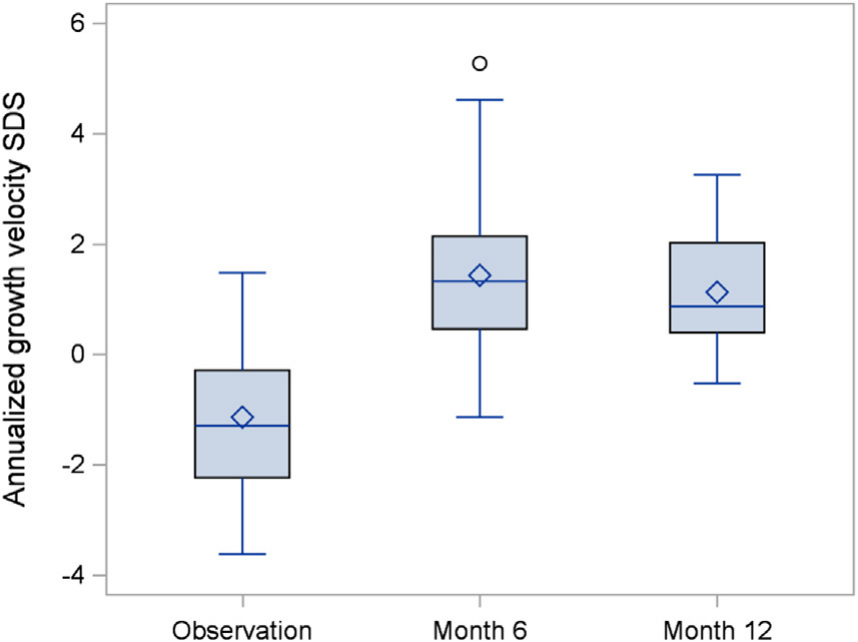
Annualized growth velocity SDS.

**Fig. 4:**
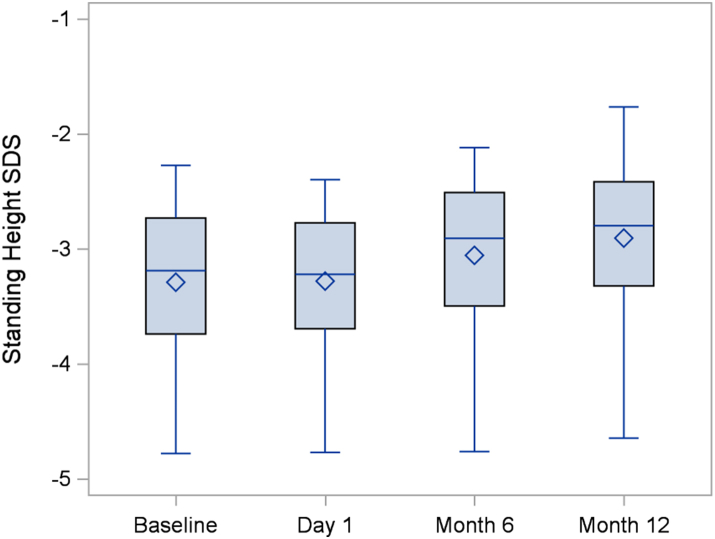
Standing height SDS.

The absolute annualized growth velocity increased from a mean of 5.12 ± 1.36 cm/year during the observation period to 6.93 ± 0.93 cm/year during the intervention period for a mean difference of 1.81 cm/year (95% confidence interval 1.16–2.46), p < 0.0001. This change corresponds to an increase in the pre-specified co-primary outcome of age and sex adjusted annualized growth velocity SDS from −1.14 ± 1.29 to +1.12 ± 1.05 (Table 2). Increases in growth velocity were evident at the Month 6 visit and were sustained to Month 12 (Figs. 2 and 3). There was no significant difference in annualized growth velocity between the Month 6 and Month 12 visit. The standing height SDS increased by a mean of 0.01 SD (95% CI: −0.06 to +0.08) during the 6-month observation period versus a mean increase of 0.37 SD (95% CI: 0.27–0.47) during the 12-month intervention period for a mean difference of 0.36 SD (95% CI: 0.26–0.47), p < 0.0001 (Table 2, Fig. 4). Using hypochondroplasia specific growth charts, the results were quite similar as the mean height SDS gain during the intervention was on average 0.38 SD (95% CI: 0.20–0.55), p < 0.0001, greater than during the observation period (Table 2, Supplementary Fig. 1). Two participants (1 male and 1 female) entered puberty during the study. Both were Tanner stage 2 at the Month 12 visit. These 2 participants had poor responses to vosoritide, and thus, the overall increase in growth velocity seen in the cohort cannot be attributed to the effects of puberty.

**Table 2:** Height efficacy outcomes.

Primary Endpoint	Observation period Mean (SD)	Treatment period Mean (SD)	Difference between treatment and observation (95% CI)	Two-sided p value
**Annualized growth velocity (cm/year)**	5.12 (1.36)	6.93 (0.93)	1.81 (1.16, 2.46)	<0.0001
**Annualized growth velocity** **SDS (cm/year)**	−1.14 (1.29)	1.12 (1.05)	2.26 (1.48, 3.05)	<0.0001

	Baseline Mean (SD)	Day 1 Mean (SD)	Month 12 Mean (SD)	Change in height SDS during observation period (95% CI)	Change in height SDS during treatment period (95% CI)	Mean difference between treatment and observation (95% CI)	Two-sided p value
**Standing height SDS**	−3.29 (0.68)	−3.28 (0.69)	−2.91 (0.68)	0.01 (−0.06, 0.08)	0.37 (0.27, 0.47)	0.36 (0.26, 0.47)	<0.0001
**Hypochondroplasia specific standing height SDS**	−0.41 (0.76)	−0.38 (0.76)	0.03 (0.82)	0.03 (−0.12, 0.19)	0.41 (0.32, 0.50)	0.38 (0.20, 0.55)	<0.0001

The observation period is the period between the baseline (screening) visit and day 1 of treatment. The intervention period is the period between day 1 and month 12 of treatment. The mean difference between treatment and observation represents the change during the treatment period minus the change during the observation period.

Secondary post-hoc analyses were performed examining the growth response based on age, baseline growth velocity and baseline height SDS (Supplementary Tables 1–4). Increases in annualized growth velocity were significant for participants aged 3 to <5 years (n = 10; p = 0.02) and 5 to <9 years (n = 11; p < 0.0001) but not 9 to <11 years (n = 3; p = 0.55) (Supplementary Fig. 2). This lack of significant increase in the oldest group may be attributable to the small sample size. The increase in annualized growth velocity in the youngest group was more significant (p = 0.003) when using the age and sex adjusted growth velocity which noted a 2.05 SD increase with treatment. Increases in growth velocity were highly significant for participants with a baseline growth velocity <5 cm/year (n = 14, p < 0.0001) but were not significant for those whose baseline growth velocity was above this cut-off (n = 10, p = 0.13). Participants with a growth velocity <5 cm/year tended to be older with only 3 of the 14 participants being <5 years old as opposed to 7 of the 10 participants with a growth velocity >5 cm/year. The sex distribution was even between the groups. Significant growth velocity increases were seen irrespective of baseline height SDS. Standing height SDS changes were significantly increased in all subgroups except for the small group of participants aged 9 and older. The hypochondroplasia specific height SDS changes showed similar patterns but were only significant in children ages 5 to <9 years old, those with a baseline growth velocity <5 cm/year, and those with a baseline height SDS <−3 SD. Additionally, the participants who did not have the Asn540Lys variant in FGFR3 had very similar growth responses to the rest of the cohort. Their changes in annualized growth velocity were 2.5 and 2.4 SD, comparable to the mean change of 2.26 SD for the entire cohort. Of the three participants previously treated with growth hormone, one had an above average response while the other two had poorer responses.

### Safety outcomes

Vosoritide was overall well tolerated with no new significant safety concerns compared to previously published reports in children with achondroplasia. No participants discontinued treatment due to a treatment related adverse event. There was one serious adverse event. A 5-year-old female developed purpura and petechiae on day 110 of vosoritide treatment. She was found to have a platelet count of <2000 and ultimately was diagnosed with post-viral immune thrombocytopenia (Grade 4). An assay for drug induced anti-platelet antibodies was performed and was negative. The participant was treated with a 5-day course of prednisolone and the platelet count returned to normal with no recurrence. Vosoritide treatment was suspended for 40 days in this participant. She restarted vosoritide treatment without incident. Despite interruption of treatment, this participant had a 2.4 cm/year increase in annualized growth velocity.

Adverse events occurring in >5% of participants in either the 6-month observation or 12-month intervention period are reported in Table 3. Injection site reactions were common with over 83% of participants reporting at least 1 injection site reaction. All injection site reactions were grade 1 or 2 and self-resolved. There were no episodes of symptomatic hypotension. One participant developed a brief episode of syncope (<10 s) on the day of her first injection. Her blood pressure was documented to be normal immediately after the syncopal episode. The participant subsequently tolerated 12 months of therapy at home with no episodes. However, she did have an episode of pre-syncope during her Month 6 visit at which time her blood pressure was also documented to be normal. As these episodes never recurred outside the hospital setting, it is unclear whether they were related to vosoritide administration or to the stress of IV placement and phlebotomy. There were no other Grade 3 or higher adverse events. Three participants had Grade 1 scoliosis noted on their Month 12 spine X-rays. Two of the three participants had scoliosis noted on their Day 1 X-rays with minimal progression at Month 12. None of the participants’ scoliosis required intervention. Three participants complained of Grade 1 dizziness. All episodes were brief and self-resolved. Of note, all episodes of dizziness occurred in participants with an underlying central nervous pathology (history of hydrocephalus, dysgenesis of corpus callosum, or foramen magnum decompression). To date, there have been no treatment related serious adverse events in any patients in the other genetic cohorts as well.

**Table 3:** Adverse events occurring in >5% of subjects.

Patients with any adverse event	Observation period (pre-treatment) # of patients (%); # of total occurrences	Treatment period (year 1) # of patients (%); # of total occurrences
**Injection related adverse events**		
Injection site erythema	N/A	13 (54.2%); 113
Injection site pain	N/A	7 (29.2%; 61)
Injection site swelling	N/A	13 (54.2%; 116)
Injection site urticaria	N/A	7 (29.2%; 19)
Injection site bruising	N/A	6 (25.0%; 20)
Any Injection site reaction (overall)	N/A	20 (83.3%; 329)
**Other adverse events**		
Abdominal pain	1 (4.2%; 7)	5 (20.8%; 15)
Cough	5 (20.8%; 6)	12 (50.0%; 20)
Diarrhea	N/A	2 (8.3%; 3)
Dizziness	N/A	3 (12.5%; 4)
Ear infection	1 (4.2%; 1)	2 (8.3%; 2)
Ear pain	2 (8.3%; 2)	N/A
Epistaxis	1 (4.2%; 1)	3 (12.5%; 3)
Fever	3 (12.5%; 9)	13 (54.2%; 29)
Headache	1 (4.2%; 7)	6 (25.0%; 17)
Laceration	1 (4.2%; 1)	3 (12.5%; 3)
Nasopharyngitis	N/A	2 (8.3%; 2)
Rash maculo-papular	2 (8.3%; 3)	2 (8.3%; 2)
Rhinorrhea	3 (12.5%; 4)	6 (25.0%; 6)
Scoliosis	N/A	3 (12.5%; 3)
Upper respiratory tract infection	N/A	2 (8.3%; 2)
Vomiting	3 (12.5%; 7)	11 (45.8%; 18)

### Secondary outcomes

**Fig. 5:**
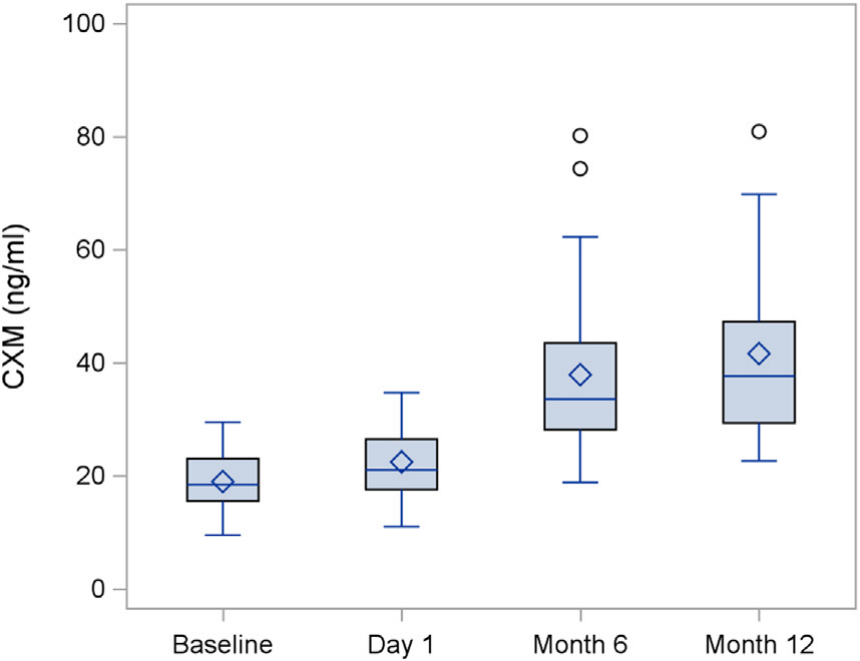
Collagen X biomarker (CXM) levels.

**Fig. 6:**
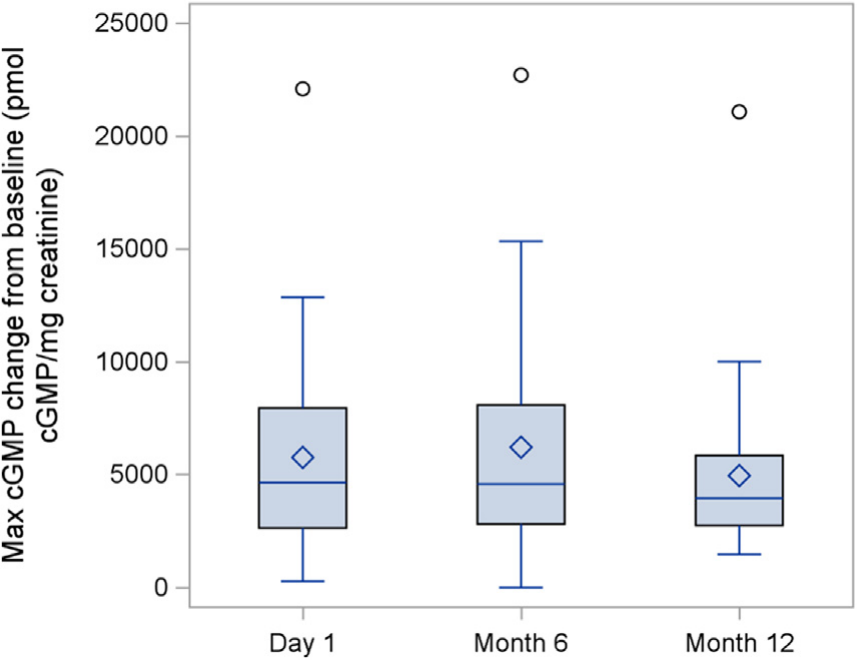
Maximum change in urine cGMP levels from baseline.

**Fig. 7:**
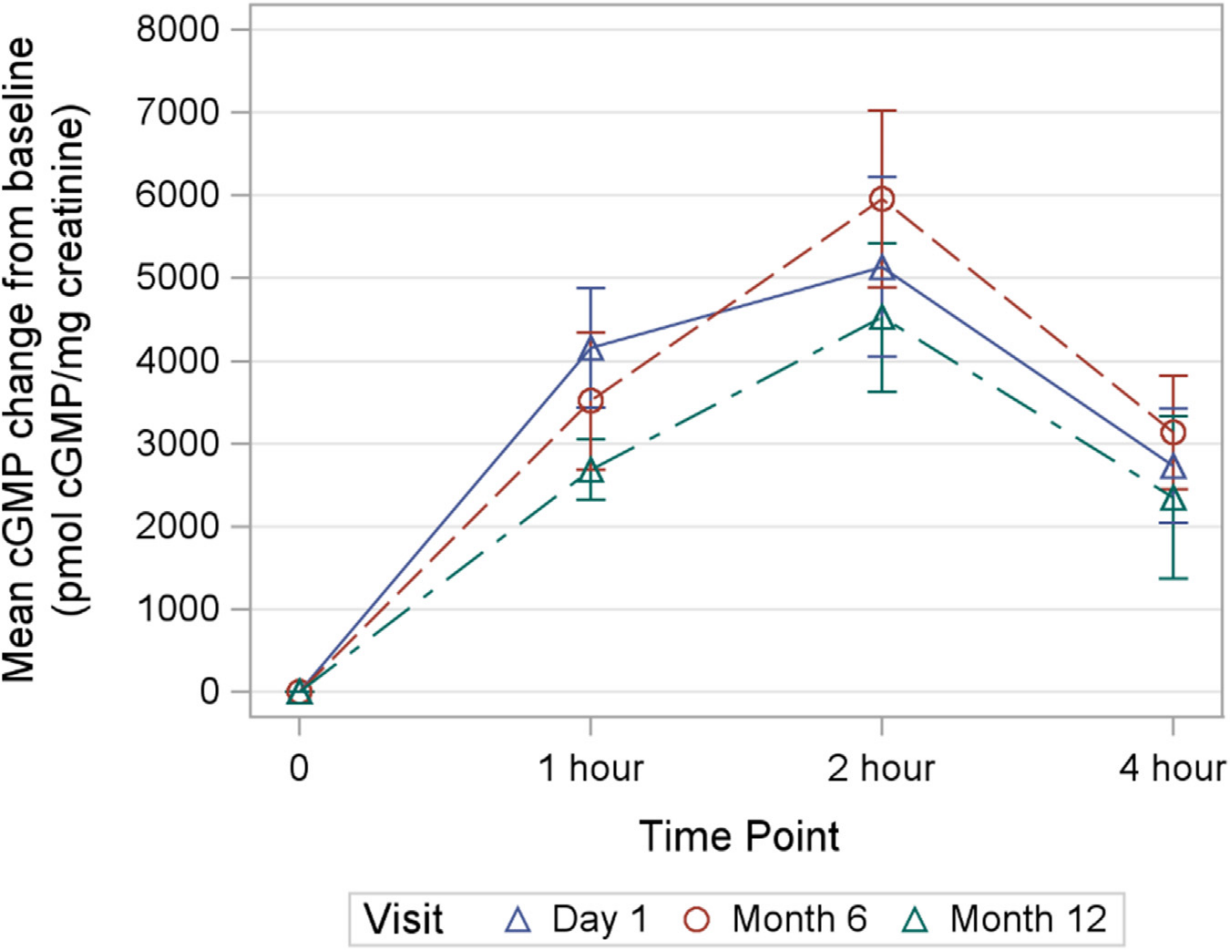
Time course of change in urine cGMP levels.

The secondary outcome results are summarized in Table 4. The bone age to chronological age ratio was unchanged over the treatment period (p = 0.67). The participants had significant disproportion at baseline with a mean sitting height to standing height ratio of +6.6 SD. The absolute sitting to standing height ratio decreased slightly over 1 year of treatment, but this was not significant when standardized for age and sex. As expected, the participants’ arm spans were on average shorter than their standing heights, but the average fell within the normal range when adjusted for sex and age. There was no significant change in this measure over the course of treatment. CXM levels increased from a baseline mean of 22.5 ± 6.5 ng/ml to 41.6 ± 15.9 ng/ml after 12 months of treatment (p < 0.0001). Increases in CXM were evident at the Month 6 visit and were sustained to Month 12 (Fig. 5). There were no significant differences in CXM levels between the Month 6 and Month 12 visit. Urine cGMP levels were measured as a biomarker of CNP activity in response to vosoritide administration. Increased in cGMP were evident within 1 h of vosoritide administration and peaked at 2 h (Fig. 6). The average maximum cGMP increase from baseline was not significantly different at Day 1, Month 6 or Month 12, indicating a sustained stimulation of CNP activity over the course of the study (Fig. 7). There were no significant changes in overall or domain specific parental-reported quality of life as measured by the QoLISSY scale (Supplementary Table 5).

**Table 4:** Summary of secondary outcomes.

Secondary endpoints	Baseline Mean (SD)	Day 1 Mean (SD)	Month 12 Mean (SD)	Change during observation period (95% CI)	Change during treatment period (95% CI)	Mean difference between treatment and observation (95% CI)	Two-sided p value
**Bone age (months)**	N/A	61.42 (30.40)	71.58 (32.17)	N/A	10.17 (7.03, 13.31)	N/A	<0.0001
**Bone age/chronological age**	N/A	0.78 (0.14)	0.79 (0.15)	N/A	0.01 (−0.03, 0.05)	N/A	0.67
**Sitting height ratio**							
Unadjusted	0.63 (0.03)	0.63 (0.02)	0.62 (0.02)	−0.001 (−0.003, 0.001)	−0.009 (−0.01, −0.006)	−0.008 (−0.012, −0.004)	0.001
Age/Sex adjusted SDS	6.60 (1.27)	6.75 (1.26)	6.59 (1.36)	0.15 (−0.11, 0.42)	−0.16 (−0.44, 0.12)	−0.31 (−0.79, 0.17)	0.20
**Arm span minus height**							
Unadjusted	−5.60 (3.27)	−6.26 (2.83)	−7.01 (3.21)	−0.66 (−1.72, 0.40)	−0.76 (−1.55, 0.04)	−0.10 (−1.74, 1.54)	0.90
Age/Sex adjusted SDS	−1.30 (1.25)	−1.58 (1.03)	−1.93 (1.14)	−0.28 (−0.68, 0.11)	−0.34 (−0.63, −0.06)	−0.06 (−0.66, 0.53)	0.83
**Collagen X Biomarker**							
CXM (ng/ml)	19.02 (5.32)	22.48 (6.49)	41.62 (15.86)	3.84 (1.94, 5.74)	17.92 (12.14, 23.69)	14.08 (7.30, 20.85)	0.0003

The observation period is the period between the baseline (screening) visit and day 1 of treatment. The intervention period is the period between day 1 and month 12 of treatment. The mean difference between treatment and observation represents the change during the treatment period minus the change during the observation period.

## Discussion

In this Phase 2 study, vosoritide demonstrated efficacy in increasing growth velocity in pre-pubertal children with hypochondroplasia. The overall safety profile was reassuring with no treatment-related serious adverse events and no participants discontinuing therapy. However, due to the small sample size, we cannot make any conclusions about rare side effects or the comparability of the safety profile to that seen in patients with achondroplasia. This is the first precision medicine therapy trial for children with hypochondroplasia and the first study of CNP-based therapy for any indication other than achondroplasia. In terms of efficacy, vosoritide increased the absolute annualized growth velocity by an average of 1.81 cm/year, similar to the increase of 1.57 cm/year that was seen in the Phase 3 study of children with achondroplasia. This increase corresponded to a 2.26 SD increase in age and sex-adjusted growth velocity. The increase in growth rate was seen irrespective of baseline height SDS but was only significant in those with baseline AGV less than or equal to 5 cm/year. It is unclear if this is due to a maximal achievable growth velocity in children with hypochondroplasia. Additionally, the absolute increase in growth velocity was lower in younger children ages 3 to <5 years as compared to those between the ages of 5–8 years. Similar findings were seen in children with achondroplasia treated with vosoritide.^15^ It is important to note that growth velocity normally decreases in children in this younger age group, so maintaining a stable growth velocity represents a positive outcome in this group. This is supported by the more significant increase in growth velocity SDS as opposed to the absolute growth velocity in the younger children.

The standing height SDS increased by 0.37 SD during the year of treatment or 0.41 SD using hypochondroplasia specific growth charts. This is slightly greater than the 0.27 SD increase seen in the Phase 3 study of children with achondroplasia. In prior studies with children with achondroplasia, the increase in growth velocity and gains in height SDS have persisted over multiple years.^16^^,^^17^ Participants in the current study with positive responses to therapy have been enrolled in an ongoing extension study to assess if this will be true in hypochondroplasia as well. It is reassuring that the bone age to chronological age ratio did not increase with treatment with vosoritide. This suggests that increases in height may lead to long-term gains in final adult height, although additional studies are needed to verify this conclusion. Urine cGMP levels, a biomarker of CNP activity, showed sustained increases after vosoritide injection over the 12 months of the study with no diminution over time. Similarly, CXM levels increased markedly with treatment and were also sustained over the 12 months of treatment. These data support the possibility of sustained growth improvements. Interestingly, increases in cGMP level were of a similar magnitude to those seen in children with achondroplasia, but increases in CXM were 3–4 times higher in the children with hypochondroplasia. This may reflect the underlying disease state and the higher overall growth velocities in children with hypochondroplasia as CXM is known to correlate with growth velocity.^14^ In the current study, no significant changes were seen in body proportions although it may take multiple years of treatment for these changes to become evident, as was the case in achondroplasia.^17^ Similarly, there was no demonstrable change in quality of life as assessed by parental survey, but these changes may also require multiple years of therapy. Participants in this study had a mean, baseline hypochondroplasia specific height SDS of −0.41 indicating that they were on average slightly shorter than the general hypochondroplasia population. This is not surprising as more severely affected children are more likely to pursue intervention.

As there are no currently approved medications for hypochondroplasia, recombinant human growth hormone (rhGH) has been used off-label in patients with hypochondroplasia with variable efficacy. To date, there are no controlled trials of rhGH in children with hypochondroplasia. A meta-analysis of 7 studies treating a total of 113 children with hypochondroplasia showed a mean first year increase in height SDS of 0.41 SD, very similar to what was seen in the current study with vosoritide.^18^ However, growth velocity slowed substantially after the first year resulting in a total increase in height of 0.61 SD after 3 years. There is no definitive data on the effect of rhGH on final adult height in individuals with hypochondroplasia. One study which treated with a higher dose of rhGH did note significant worsening of body disproportion after three years of treatment.^19^

In conclusion, 1 year of daily administration of vosoritide resulted in improved growth in children with hypochondroplasia with a relatively benign side effect profile. Additional studies are needed to see the long-term effects of vosoritide in children with hypochondroplasia. This study supports further development of CNP analog therapy for children with hypochondroplasia.

## Contributors

AD conceptualized the study, wrote the protocol, conducted study visits, and wrote the first draft of the manuscript. AZ did the statistical analysis. RKS, KB, TM, MGC, NS, RS, ND and NM conducted study visits and assisted with patient management during the trial. AD and AZ accessed and verified the data as reported, and vouch for adherence of the trial to the protocol and complete reporting of all adverse events. All authors reviewed the manuscript.

## Data sharing statement

The de-identified individual participant data that underlie the results reported in this Article (including text, tables, figures, and supplement) will be made available upon request to the corresponding author together with the research protocol, for non-commercial, academic purposes. Requests must include a research proposal clarifying how the data will be used, including proposed analysis methodology. A data use agreement with Children's National Hospital will be required prior to data transfer.

## Declaration of interests

AD and NM have served as consultants for BioMarin, but all compensation has been paid to Children's National Hospital and neither author has received any personal compensation from BioMarin. AD received an investigator-initiated grant from BioMarin to fund the current study. RKS has received an investigator-initiated grant from BioMarin to fund a study of vosoritide in girls with Turner syndrome. The remaining authors have nothing to disclose.

## Appendix A Supplementary data

Supplementary Material

Vosoritide Protocol
