# Supplementary material for: Vosoritide treatment for children with hypochondroplasia: a phase 2 trial
Source: eClinicalMedicine. 2024 Apr 11;71:102591. doi: 10.1016/j.eclinm.2024.102591 (PMC11133798; doi:10.1016/j.eclinm.2024.102591)
Supplement: Vosoritide Protocol [file mmc2.pdf]

VOSORITIDE FOR SELECTED GENETIC CAUSES OF SHORT STATURE

Protocol Number: Pro00013585

National Clinical Trial (NCT) Identified Number: NCT04219007

Principal Investigator: Andrew Dauber, MD MMSc

IND Sponsor: Andrew Dauber, MD MMSc

Funded by: BioMarin

Version Number: v.2.9

2 May 2023

Table of Contents

STATEMENT OF COMPLIANCE 1
1 PROTOCOL SUMMARY 1
1.1 Synopsis 1
1.2 Schema 4
1.3 Schedule of Activities (SoA) 7
2 INTRODUCTION 11
2.1 Study Rationale 11
2.2 Background 11
2.3 Risk/Benefit Assessment 11
2.3.1 Known Potential Risks 19
2.3.2 Known Potential Benefits 20
2.3.3 Assessment of Potential Risks and Benefits 20
3 OBJECTIVES AND ENDPOINTS 20
4 STUDY DESIGN 23
4.1 Overall Design 23
4.2 Scientific Rationale for Study Design 23
4.3 Justification for Dose 24
4.4 End of Study Definition 24
5 STUDY POPULATION 24
5.1 Inclusion Criteria 24
5.2 Exclusion Criteria 27
5.3 Lifestyle Considerations 27
5.4 Screen Failures 28
5.5 Strategies for Recruitment and Retention 28
6 STUDY INTERVENTION 28
6.1 Study Intervention(s) Administration 29
6.1.1 Study Intervention Description 29
6.1.2 Dosing and Administration 29
6.2 Preparation/Handling/Storage/Accountability 30
6.2.1 Acquisition and accountability 30
6.2.2 Formulation, Appearance, Packaging, and Labeling 31
6.2.3 Product Storage and Stability 31
6.2.4 Preparation 31
6.3 Measures to Minimize Bias: Randomization and Blinding 32
6.4 Study Intervention Compliance 32
6.5 Concomitant Therapy 32
6.5.1 Rescue Medicine 32
7 STUDY INTERVENTION DISCONTINUATION AND PARTICIPANT DISCONTINUATION/WITHDRAWAL 32
7.1 Discontinuation of Study Intervention 32
7.2 Participant Discontinuation/Withdrawal from the Study 34
7.3 Lost to Follow-Up 35
8 STUDY ASSESSMENTS AND PROCEDURES 35
8.1 Efficacy Assessments 35
8.2 Safety and Other Assessments 37

8.3 Adverse Events and Serious Adverse Events 39
8.3.1 Definition of Adverse Events (AE) 39
8.3.2 Definition of Serious Adverse Events (SAE) 40
8.3.3 Classification of an Adverse Event 40
8.3.4 Time Period and Frequency for Event Assessment and Follow-Up 41
8.3.5 Adverse Event Reporting 42
8.3.6 Serious Adverse Event Reporting 42
8.3.7 Reporting Events to Participants 43
8.3.8 Events of Special Interest 43
8.3.9 Reporting of Pregnancy 43
8.4 Unanticipated Problems 43
8.4.1 Definition of Unanticipated Problems (UP) 43
8.4.2 Unanticipated Problem Reporting 43
8.4.3 Reporting Unanticipated Problems to Participants 44
9 STATISTICAL CONSIDERATIONS 44
9.1 Statistical Hypotheses 44
9.2 Sample Size Determination 44
9.3 Populations for Analyses 45
9.4 Statistical Analyses 45
9.4.1 General Approach 45
9.4.2 Analysis of the Primary Efficacy Endpoint(s) 45
9.4.3 Analysis of the Secondary Endpoint(s) 45
9.4.4 Safety Analyses 45
9.4.5 Baseline Descriptive Statistics 46
9.4.6 Planned Interim Analyses 46
9.4.7 Sub-Group Analyses 46
9.4.8 Tabulation of Individual participant Data 46
9.4.9 Exploratory Analyses 46
10 SUPPORTING DOCUMENTATION AND OPERATIONAL CONSIDERATIONS 46
10.1 Regulatory, Ethical, and Study Oversight Considerations 46
10.1.1 Informed Consent Process 46
10.1.2 Study Discontinuation and Closure 47
10.1.3 Confidentiality and Privacy 48
10.1.4 Future Use of Stored Specimens and Data 48
10.1.5 Key Roles and Study Governance 49
10.1.6 Safety Oversight 49
10.1.7 Clinical Monitoring 49
10.1.8 Quality Assurance and Quality Control 50
10.1.9 Data Handling and Record Keeping 50
10.1.10 Protocol Deviations 51
10.1.11 Publication and Data Sharing Policy 51
10.1.12 Conflict of Interest Policy 51
10.2 Additional Considerations 52
10.3 Abbreviations 52
10.4 Protocol Amendment History 54

11 REFERENCES 56

STATEMENT OF COMPLIANCE

The trial will be conducted in accordance with International Conference on Harmonization Good Clinical Practice (ICH GCP), applicable United States (US) Code of Federal Regulations (CFR), and the BioMarin Terms and Conditions of Award. The Principal Investigator will assure that no intentional deviation from, or significant changes to the protocol will take place without prior agreement from the funding agency and documented approval from the Institutional Review Board (IRB), except where necessary to eliminate an immediate hazard(s) to the trial participants. All personnel involved in the conduct of this study have completed Human Subjects Protection and ICH GCP Training.

The protocol, informed consent form(s), recruitment materials, and all participant materials will be submitted to the IRB for review and approval. Approval of both the protocol and the consent form must be obtained before any participant is enrolled. Any amendment to the protocol will require review and approval by the IRB before the changes are implemented to the study. All changes to the consent form will be IRB approved; a determination will be made regarding whether a new consent needs to be obtained from participants who provided consent, using a previously approved consent form.

1 PROTOCOL SUMMARY

1.1 SYNOPSIS

Title: Vosoritide for Selected Genetic Causes of Short Stature

Study Description: Short stature can be caused by a number of different genetic defects, many of which directly affect the growth plates. Vosoritide, a C-type natriuretic peptide (CNP) analog, targets chondrocytes within the growth plate leading to increased cell proliferation and hypertrophy. We hypothesize that patients with selected genetic causes of short stature will respond to vosoritide treatment leading to increased growth velocity. This study will enroll patients with selected genetic causes of short stature for whom evidence exists to support the potential for improved growth. The subjects will be followed for a 6 month observation period to obtain baseline growth velocity, safety profiles and quality of life assessments. Subjects will then be treated with vosoritide for 12 months and will be assessed for safety monitoring and improvement in height outcomes. Subjects with a positive response to therapy will be given the option to continue in the extension phase of the study during which they will continue to receive vosoritide until growth cessation.

Objectives:

The primary objectives of the study are as follows:

- To evaluate the safety and tolerability of daily subcutaneous (SC) injections of vosoritide administered for 12 months in patients with selected genetic causes of short stature
- To evaluate the change from baseline in age-sex standardized annualized growth velocity after 12 months of daily SC injections of vosoritide in patients with selected genetic causes of short stature

- To evaluate the change from baseline in age-sex standardized height standard deviation score (SDS) after 12 months of daily SC injections of vosoritide in patients with selected genetic causes of short stature

The secondary objectives of the study are as follows:

- To evaluate changes in body proportions from baseline after 12 months of daily SC injections of vosoritide
- To evaluate changes from baseline in bone age/chronological age after 12 months of daily SC injections of vosoritide

The exploratory objectives of the study are as follows:

- To evaluate the pharmacokinetic (PK) profile of vosoritide in patients with selected genetic causes of short stature
- To evaluate changes from baseline in bone mineral density (BMD) after 12 months of daily SC injections of vosoritide
- To evaluate changes from baseline in vosoritide activity biomarkers and bone/collagen biomarkers after 12 months of daily SC injections of vosoritide
- To evaluate immunogenicity and assess impact on safety and efficacy measures after 12 months of daily SC injections of vosoritide
- To explore the relationship between genetic etiology and safety and efficacy
- To evaluate changes from baseline in quality of life using the QoLISSY scale after 12 months of daily SC injections of vosoritide

During the extension study, all of the primary, secondary and exploratory aims will remain the same, but the timeframe will be extended from 12 months until growth cessation or subject withdrawal from the study.

Endpoints:

The primary study endpoints include:

- Incidence of adverse events per subject with particular attention to Grade 3 and 4 adverse events as well as to rates of hypotension
- Change from baseline in age-sex standardized annualized growth velocity after 12 months of treatment
- Change from baseline in age-sex standardized height SDS after 12 months of treatment

The secondary study endpoints include:

- Change from baseline in seated height ratio after 12 months of treatment
- Change from baseline in arm span minus standing height after 12 months of treatment
- Change from baseline in bone age/chronological age after 12 months of treatment

During the extension study, all of the endpoints will remain the same, but the timeframe will be extended from 12 months until growth cessation or subject withdrawal from the study.

Study Population:

Accrual Ceiling:

The study will include up to 75 pre-pubertal children ages ≥3 years 0 days AND ≤10 years 364 days for males, ≤9 years 364 days for females with selected genetic causes of short stature (as defined below). Children will be recruited throughout the United States and will be brought to Children's National Hospital for all study visits.
We estimate screening up to 100 subjects to achieve the feasible sample size for the trial.

Phase: I/II

Description of Sites/Facilities Enrolling Participants:

This is a single center study at Children's National Hospital. Recruitment will occur throughout the United States, and patients will be brought to Children's National Hospital for all study visits.

Description of Study Intervention:

This is a pediatric, Phase I/II, open-label single dose study of up to 75 subjects with selected genetic causes of short stature. Subjects who are 3 to 10 years old inclusive for males and 9 years old inclusive for females who have genetic defects in one of the categories defined in the inclusion criteria and meet all eligibility requirements will participate. After enrollment, subjects will be followed for a 6 month observation only period to establish a baseline height velocity as well as safety profile and quality of life assessment. Vosoritide will then be administered daily via subcutaneous injection at a dose of 15 µg/kg/day for 12 months. Subjects with a positive response (as defined in the inclusion criteria for extension phase) to therapy will be given the option to continue in the extension phase of the study during which they will continue to receive vosoritide until growth cessation or subject withdrawal from the study.

PK sampling as well as safety monitoring will be conducted for all subjects on the first day that the study drug is received. The safety monitoring includes a minimum of 8 hours post-dose observation on the first day of dosing. Vital signs will be monitored frequently on that day, including approximately every 10-20 minutes for the first 2 hours post dose. Additional assessments include anthropometric measurements, physical examination, laboratory monitoring, ECG, DXA scan, bone age and spine X-rays. On day 2, the subject will return to the clinical research center for their second injection as well as 2 hours of post-injection monitoring. On days 3-5, subjects will receive a daily telephone call to review symptoms and issues with medication administration. After day 5, phone calls will occur on a bi-weekly schedule in-between study visits for months 1-5. During all telephone calls, adverse events as well as medication administration compliance will be assessed.

Subjects will return to Children's National Hospital for visits at 6 and 12 months. At those visits, anthropometric and safety assessments will be obtained as well as additional PK data at the 6 and 12 month visit.

Additional assessments noted in the prior paragraph will be repeated at the 6 and 12 month visits as detailed in the protocol. The dose of medication will be adjusted for weight at the 6 month visit if the subject has experienced weight changes.

During the extension phase, subjects will continue to be seen at Children's National every 6 months until growth cessation or subject withdrawal. Telephone visits will occur at the 3-month time point mid-way between each study visit.

Study Duration: 36 months; Extension phase of study may last up to an additional 15 years

Participant Duration: 18 months; Extension phase may last up to ~15 years if a subject starts at age 3 years and stops growing at age 18 years

1.2 SCHEMA

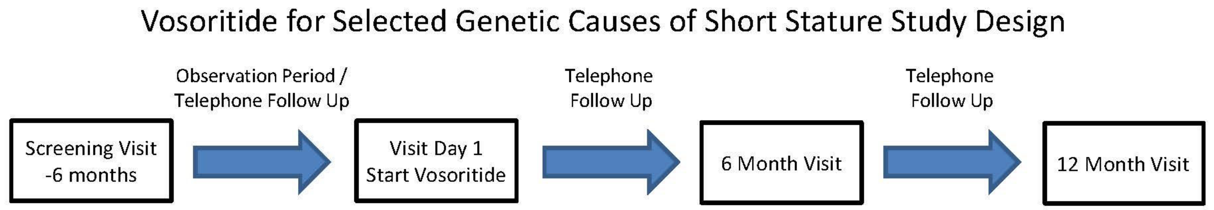

-6 Months | Screening and Initiation of Observation Period

- Total n=up to 100
- Obtain informed consent
- Screen potential participants by inclusion and exclusion criteria
- Obtain history
- Obtain baseline physical exam and anthropometric measurements
- Obtain baseline screening labs, quality of life survey, and diagnostic studies
- Details provided in **Section 1.3, Schedule of Activities**

Months -5 through -1 | Monthly phone calls

- Review health events
- Assess for adverse events/concomitant medications

Visit Day 1 (6months post screening) | Vosoritide Initiation

- Intervention Group (n= up to 75)
- Initiate vosoritide treatment
- PK sampling
- Repeat laboratory, quality of life survey and diagnostic imaging evaluations, see **Section 1.3, Schedule of Activities**

Visit Day 2 | Vosoritide Administration and Observation for Adverse Events

- Caregiver demonstrates proper medication administration in the clinic
- Assess for adverse events

Days 3 through 5 | Daily Phone Calls

- Review medication administrations
- Review health events
- Assess for adverse events/concomitant medications

Months 1 through 5 | Bi-weekly Phone Calls

- Review medication administrations
- Review health events
- Assess for adverse events/concomitant medications

6 Month Visit | Follow-up assessments of study endpoints and safety

- Obtain repeat quality of life survey, physical exam and anthropometric measurements
- Obtain repeat screening labs, PK sampling and diagnostic studies, see **Section 1.3, Schedule of Activities**

Months 7 through 11 | Monthly Phone Calls

- Review medication administrations
- Review health events
- Assess for adverse events/concomitant medications

12 Month Visit | Follow-up assessments of study endpoints and safety

- Obtain repeat quality of life survey, physical exam and anthropometric measurements
- Obtain repeat screening labs, PK sampling, and diagnostic studies, see **Section 1.3, Schedule of Activities**

EXTENSION PHASE DESIGN

The extension phase will begin immediately after the 12-month visit. Eligible subjects will sign an addendum to the informed consent which outlines the details of participation in the extension study. Subjects will be seen in-person at Children's National every 6 months (+/- 2 weeks). Activities at the in-person study visits will include:

- Anthropometric Measurements
- Physical Exam
- Tanner Staging
- Vital Signs
- Quality of Life Survey
- Screening Labs
- Collagen X Marker sampling
- Vosoritide dose adjustment
- Review health events, concomitant medications, and medication administration
- Bone age X-ray – every 12 months
- DXA scan – every 24 months
- Additional safety imaging as needed due to clinical concern or prior abnormality

Additionally, subjects will have a telephone visit at the 3-month time point (+/- 2 weeks) between in-person visits. Activities at the telephone study visits will include:

- Review medication administration
- Review health events
- Assess for adverse events/concomitant medications

1.3 SCHEDULE OF ACTIVITIES (SOA)

Procedure	Screening and Initiation of Observation (-6 months)	Monthly Phone calls +/- 4 days (Months -5 through -1)	Day 1 visit (6 months +/- 2 weeks after screening)	Day 2 Visit	Days 3 through 5 Followed by Bi-weekly Phone calls +/- 4 days (Months 1 through 5)	6 month visit (+/- 2 weeks)	Monthly Phone calls +/- 4 days (Months 7 through 11)	12 month visit (+/- 2 weeks)	Early termination
Informed consent	X								
Medical History	X		X						
Parental anthropometrics[a]	X								
Physical examination including a complete musculoskeletal exam	X		X			X		X	X
Tanner stage	X		X			X		X	X
Vital signs[b]	X		X	X		X		X	X
Anthropometric measurements[c]	X		X			X		X	X
ECG	X		X			X		X	X
Echocardiogram	X							X	X
Clinical labs[d]	X		X			X		X	X
Tissue Transglutaminase Antibody	X								
Anti-vosoritide immunogenicity[e]			X			X		X	X
PK sampling[f]			X			X		X	
PD markers[g]	X		X			X		X	X
DXA scan[h]			X	*				X	X
Bone Age X-ray			X					X	X
Spine X-ray[i]			X	*		*		X	X

QoLISSY questionnaires	X		X			X		X	X
Health events reviews		X	X	X	X	X	X	X	X
Concomitant medications	X	X	X	X	X	X	X	X	X
Study drug administration review			X	X	X	X	X	X	X
Adverse event reviews	X	X	X	X	X	X	X	X	X

[a] Both parents heights should be measured if available.

[b] All treatment visits have pre-dose vital sign assessments. Vital signs include: body temperature in degrees Celsius (°C), heart rate, BP, and respiratory rate. On visit Day 1 only, minimum assessment frequency is every 15 min (± 5 min) for 2 hours post dose; and assessment is taken every 30 min (±5 min) from 2 to 4 hours post dose. From 4 to 8 hours post-dose, measurements are taken every 60 min (± 10 min). At the Day 2 Visit, vital signs will be assessed every 30 minutes (± 5 min) for 2 hours post dose. Vital sign measurements are taken in a sitting position after at least 5 minutes of rest. Heart rate should be taken at each timepoint that BP is measured. When blood samples and BP assessments are scheduled at the same time or within the same time window, BP should be measured before blood samples are drawn. Vital signs may be monitored more frequently or for longer duration post-dose as clinically indicated.

[c] Anthropometric measurements include standing height, weight, sitting height, head circumference and arm span. Standing height should be measured 5 times using a calibrated stadiometer. Sitting height should be measured three times. Weight should be measured on a digital scale.

[d] Clinical labs include: complete blood count with differential, complete metabolic panel, lipid panel, TSH, free T4, IGF-I, IGFBP-3, prolactin. All labs will be obtained through the clinical lab at Children's National Hospital. Additionally, an extra 10 cc of blood will be obtained for storage for future analysis.

[e] Antibodies: Total anti-BMN 111 immunogenicity analysis (TAb) and neutralizing antibodies (NAb) will be collected from serum. TAb will be measured on all samples as indicated in the table. NAb will be stored and testing of NAb will only be triggered when a decline in cGMP signal is observed in the cGMP biomarker assay at both the 6 and 12 month visits in a TAb positive subject.

[f] PK: On Day 1 and months 6 and 12, PK plasma samples are collected pre-dose and at 5 (± 2 min), 15 (± 2 min), 30 (± 5 min), 60 (± 5 min), 90 (± 5 min), 120 (± 5 min), and 180 (± 5 min) min post-dose.

[g] PD: Samples will be obtained for urinary cGMP at baseline at the Screening Visit and at baseline and 1 hour, 2 hours and 4 hours post vosoritide injection at the Day 1 Visit, 6 Month Visit and 12 Month Visit. Urine creatinine samples will be obtained at all of the same time points. Serum collagen X marker measurement will be obtained at baseline at the Screening Visit, Day 1 Visit, 6 Month Visit and 12 Month Visit.

[h] DXA scan will include whole body less head as well as a lumbar spine DXA. If the DXA is unable to be completed on Day 1, it may be completed on Day 2.

[i] Spine X-rays will include an posteroanterior view of the spine. If the spine X-ray is unable to be completed on Day 1, it may be completed on Day 2. If the clinical examination suggests a new onset spine asymmetry, an additional spine X-ray will be performed at the 6 month visit.

Schedule of Activities for Extension Phase of Study

Procedure	Extension Study Initiation Visit (occurs on same day as 12-month visit)	3-Month Phone Visit +/- 2 weeks	In-person Visit (6 months +/- 2 weeks after prior in-person visit)	3-Month Phone calls +/- 2 weeks	In-person Visit (6 months +/- 2 weeks after prior in-person visit)	Early termination
Assess Eligibility	X					
Informed Consent	X					
Physical examination including a complete musculoskeletal exam			X		X	X
Tanner stage			X		X	X
Vital signs[a]			X		X	X
Anthropometric measurements[b]			X		X	X
Clinical labs[c]			X		X	X
PD marker[d]			X		X	X
DXA scan[e]					X*	X
Bone Age X-ray					X	X
Additional Safety Imaging[f]			X		X	X
QoLISSY questionnaires			X		X	X
Health events reviews		X	X	X	X	X
Concomitant medications		X	X	X	X	X
Study drug administration review		X	X	X	X	X
Adverse event reviews		X	X	X	X	X
Dose Adjustment[g]			X		X	

Note: The schedule for the extension phase repeats every year with alternating telephone and in-person visits every 3 months. [a] All treatment visits have pre-dose vital sign assessments. Vital signs include: body temperature in degrees Celsius (°C), heart rate, BP, and respiratory rate. Vital sign measurements are taken in a sitting position after at least 5 minutes of rest. Vital signs should be measured before blood samples are drawn. Vital signs may be monitored more frequently or for longer duration post-dose as clinically indicated.

[b] Anthropometric measurements include standing height, weight, sitting height, head circumference and arm span. Standing height should be measured 5 times using a calibrated stadiometer. Sitting height should be measured three times. Weight should be measured on a digital scale.

[c] Clinical labs include: complete blood count with differential, complete metabolic panel, lipid panel, TSH, free T4, IGF-I, IGFBP-3, prolactin. All labs will be obtained through the clinical lab at Children's National Hospital. Additionally, an extra 10 cc of blood will be obtained for storage for future analysis.

[d] PD: Serum collagen X marker measurement will be obtained at all in-person visits.

[e] DXA scan will include whole body less head as well as a lumbar spine DXA. It will be done every 24 months.

[f] Additional safety imaging may include echocardiograms, spine X-rays or any musculoskeletal imaging that is needed based on a clinical concern of the investigator.
[g] Vosoritide dose will be adjusted for weight at every in-person visit to maintain the prescribed dose of 15 µg/kg/day

2 INTRODUCTION

2.1 STUDY RATIONALE

It is becoming increasingly understood that genetic factors play a major role in determining an individual's height. Recent research, detailed below, suggests that many patients who present with short stature likely have genetic defects in genes involved in growth plate physiology. In current clinical care, many of these patients are classified as having idiopathic short stature. While growth hormone is approved for patients with idiopathic short stature, many insurance companies do not cover this diagnosis and the response to therapy in patients is quite variable and often quite modest [1]. BioMarin recently developed a new pharmaceutical agent, vosoritide, which acts as a C-type natriuretic peptide (CNP) analog and directly stimulates the growth plate. A Phase II study of this medication in patients with achondroplasia, a severe growth plate disorder, demonstrated efficacy at increasing growth velocity with an excellent safety profile [2]. We hypothesize that patients with selected causes of short stature will benefit from treatment with vosoritide. The selected causes included in this study all interact with the CNP pathway and there is evidence to support a prospect of benefit with vosoritide for children with these genetic causes of short stature. This study will enroll patients with selected genetic causes of short stature (defined in the inclusion criteria) and will follow them for a 6 month observation period to obtain a baseline growth velocity, safety profile, and quality of life assessment. Patients will then be treated with vosoritide for 12 months and will be assessed for safety and tolerability as well as improvement in height outcomes. Subjects with a positive response to therapy will be given the option to continue in the extension phase of the study during which they will continue to receive vosoritide until growth cessation.

2.2 BACKGROUND

Children with short stature often present to the pediatric endocrinologist for medical evaluation. After elimination of an underlying chronic medical illness or hormonal deficiency, many of the patients' growth impairments are attributed to an underlying genetic predisposition to short stature. In recent years, advances in genomic technologies have allowed us to dissect the genetic underpinnings of short stature [3,4]. Many of the genetic etiologies are centered on defects in the growth plate which is the primary site of action responsible for linear growth in humans [5]. In perhaps the most comprehensive study to date examining the yield of genetic diagnosis in short stature, Hauer et al. found that heterozygous carriers of recessive skeletal dysplasia genes represented 3.5% of all patients presenting for evaluation with short stature [6]. In the group of patients who underwent exome sequencing, 2.5% had mutations in *ACAN* (the gene for Aggrecan, a proteoglycan found in the extracellular matrix of the growth plate) and 1.5% had mutations in *NPR2*, the gene encoding the NPR-B receptor for C-type natriuretic peptide (CNP) [6]. In a recent study from the Czech Republic, 33 patients with familial short stature who were being treated with growth hormone were studied to identify a genetic etiology [7]. Seventeen of the 33 (52%) had a likely genetic etiology identified with 9 of 17 (53%) being attributed to variants in growth plate related genes [7]. Current therapeutic options to treat patients with genetic growth disorders are quite limited. Growth hormone has been approved for a limited number of indications but response to growth hormone is quite variable. Therefore, there is a need to explore novel therapeutic approaches to genetic causes of short stature.

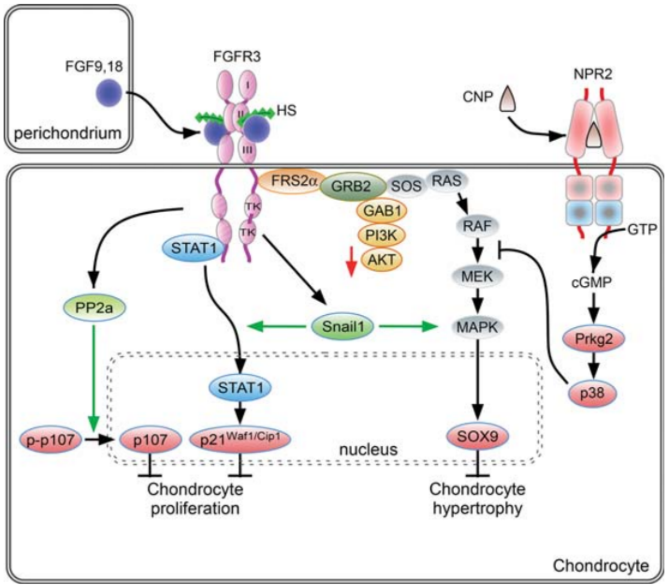

Figure taken from Reference [8]

BioMarin has developed a drug called vosoritide which is a modified recombinant CNP that is administered as a daily subcutaneous injection. Vosoritide has initially been studied in patients with achondroplasia, a condition due to an activating mutation in *FGFR3* which leads to severe perturbations in growth plate physiology. Achondroplasia was targeted due to the clear interaction between the CNP/NPR-B signaling pathway and the FGFR3 pathway. Activating mutations in FGFR3 lead to increased downstream signaling via SOS > RAS > RAF-1 ultimately increasing signaling in the MEK/ERK pathway. Increased phosphorylation of ERK1/2 is an important marker of increased activity of this pathway and ultimately leads to decreased chondrocyte proliferation and hypertrophy. CNP binds to its receptor, NPR-B, and generates cGMP, which activates a number of different signaling mediators including cGMP-dependent protein kinases. One of these kinases, protein kinase G, inhibits the activation of RAF-1, and ultimately leads to decreased activation of ERK1/2. Thus, this inhibitory effect of CNP on the FGFR3 pathway leads to an increase in chondrocyte proliferation and differentiation and an increase in cartilage matrix synthesis [8].

Results of BioMarin's Phase II study were recently published in the *New England Journal of Medicine* [2]. In this Phase II study, they treated 35 patients with achondroplasia in a dose escalation study of vosoritide with doses ranging from 2.5 µg/kg to 30 µg/kg. First, they showed a very reassuring safety profile. Four patients had serious adverse events (grade 3 obstructive sleep apnea, grade 1 tonsillar hypertrophy, grade 3 thyroglossal duct cyst, and grade 3 syrinx – each in 1 patient), all of which were likely associated with the underlying achondroplasia. Only a single patient had a symptomatic hypotensive episode on day 281 of the extension study which self-resolved. The remainder of the safety profile was quite benign with some mild local injection site reactions. Patients receiving the highest two doses of vosoritide had a sustained increase in annualized growth velocity although there was no difference seen between the 15 and 30 µg/kg groups. Over the course of the study, the patients treated with 15 µg/kg achieved a mean gain in height standard deviation score of 1.03 SD. Details of all of the pre-clinical and clinical development data for vosoritide are included in the Investigator's Brochure which accompanies this protocol [9].

These Phase II results show proof of principle that patients with a severe defect in a chondrocyte signaling pathway (i.e. activating mutation in *FGFR3* causing achondroplasia) can have increases in growth velocity when treated with vosoritide. It is currently unknown how patients with other causes of short stature will respond to vosoritide. Our hypothesis is that patients with selected genetic causes of

short stature, in particular those with evidence suggesting that they affect the same signaling pathways, will also respond to vosoritide treatment with an increase in growth velocity. Furthermore, it is plausible that these patients will have more robust growth responses than those with achondroplasia as achondroplasia is an extremely severe perturbation of chondrocyte physiology.

In the current protocol, we now seek to extend this prior work into a new cohort of patients who have other genetic etiologies for short stature for which there is evidence to support the potential for benefit with vosoritide. Each of the 6 categories below leads to a pathological increase in ERK1/2 phosphorylation and thus should be amenable to treatment with vosoritide. Specifically, this protocol targets patients falling into the following categories:

1. CNP deficiency due to mutations in the *NPPC* gene
2. Hypochondroplasia due to mutations in the *FGFR3* gene
3. Patients with heterozygous defects in *NPR2* (the gene that encodes NPR-B)
4. Rasopathy patients (including Noonan syndrome, Costello syndrome, Cardiofaciocutaneous syndrome, Neurofibromatosis Type 1)
5. Patients with SHOX deficiency (not including Turner Syndrome)
6. Patients with heterozygous defects in *ACAN* (the gene that encodes Aggrecan)

Below is the evidence to support the prospect of direct benefit in each of these categories.

<u>CNP deficiency due to mutations in the NPPC gene</u>

To date, 2 families have been identified who have heterozygous mutations in the CNP gene itself (*NPPC*) presenting with dominantly inherited short stature with heights ranging from -4.3 to -2.3 SD [10]. The affected patients were noted to have small hands as well but were otherwise healthy. Both mutations were missense variants (p.Arg117Gly and p. Gly119Cys) affecting highly conserved amino acids in the CNP ring. In vitro studies showed that these missense variants led to decreased cGMP production upon stimulation of the NPR-B receptor in both the homozygous and heterozygous state [10].

Patients with CNP deficiency in either the heterozygous or homozygous state are natural candidates for vosoritide as CNP replacement therapy. As further evidence to support this concept, Fujii et al. crossed a CNP knockout mouse model (*Nppc*$^{-/-}$) with a mouse overexpressing a transgene for CNP. The *Nppc*$^{-/-}$ mice demonstrate decreased body length, bone size, and growth plate width. After crossing with the transgenic mice, all of these phenotypic features were greatly improved suggesting that circulating CNP is able to rescue the phenotype of global CNP deficiency [11].

<u>Hypochondroplasia</u>

Hypochondroplasia is due to milder mutations in the *FGFR3* gene with the p.Asn540Lys mutation being by far the most common cause [12]. The rationale for using vosoritide in hypochondroplasia is primarily an extension of the positive clinical trial results in patients with achondroplasia, a more severe perturbation of the same gene. As noted earlier, in the Phase II trial, patients with achondroplasia treated with 15 µg/kg of vosoritide achieved a mean gain in height standard deviation score of 1.03 SD [2]. Additionally, there have been multiple mouse studies of mice with *FGFR3* mutations representing either thanataphoric dysplasia [13] or achondroplasia that have responded to CNP/vosoritide treatment with improved growth [14,15].

In vitro studies have shown that the p.Asn540Lys mutation causing hypochondroplasia leads to increased pERK (a marker of activation of the FGFR3 pathway) and decreased cell proliferation [16]. Furthermore, in unpublished data, BioMarin has replicated this data in rat chondrosarcoma cells transfected with FGFR3 containing the hypochondroplasia mutation. Furthermore, the data show that addition of vosoritide to these cell lines leads to decrease ERK1/2 activation similar to what is seen in achondroplasia (see figure below). This provides strong support to the notion that patients with hypochondroplasia should respond to vosoritide.

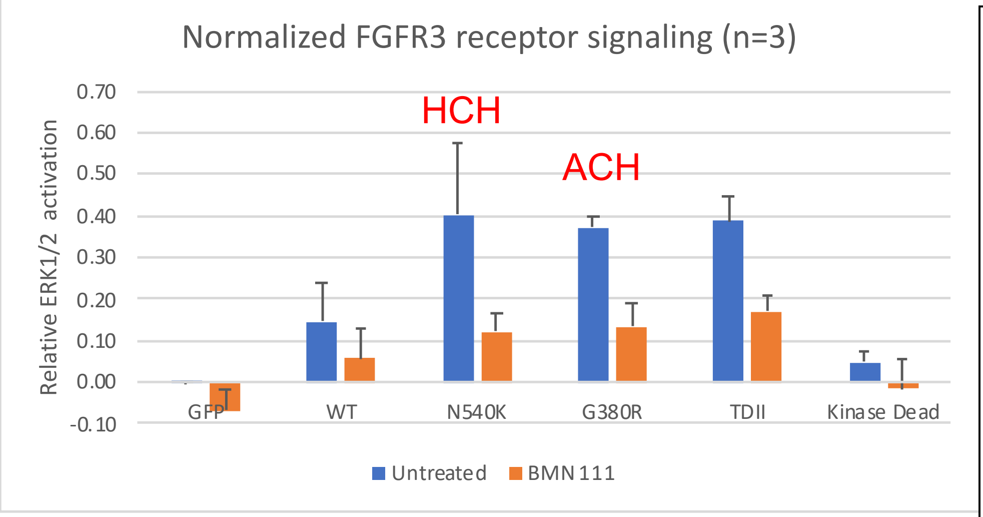

Figure includes internal unpublished BioMarin data. Mutations causing Hypochondroplasia (HCH) and Achondroplasia (ACH) are noted. Error bars represent three independent experiments.

<u>Patients with heterozygous defects in *NPR2* (the gene that encodes NPR-B)</u>

As discussed above, *NPR2* encodes the NPR-B receptor for CNP found on chondrocytes. When activated, NPR-B begins a signaling cascade that inhibits FGFR3 signaling resulting in decreased ERK activity, ultimately promoting chondrocyte proliferation and hypertrophy. Homozygous loss-of-function mutations in *NPR2* cause the severe acromesomelic dysplasia Maroteaux type [17]. In 2006, Olney et al. reported that heterozygous carriers of *NPR2* mutations presented with isolated short stature without significant syndromic features or skeletal abnormalities [18]. Subsequent to this report, a number of groups have examined the rate of *NPR2* mutations in patients with idiopathic short stature. Vasques et al found mutations in *NPR2* in 3 out of 47 patients presenting with idiopathic short stature [19]. Amano et

al identified mutations in 2 of 101 Japanese patients with short stature [20], and Hisado-Oliva et al found that 3% of 197 patients with disproportionate short stature had mutations in *NPR2* [21]. Our group identified pathogenic mutations in *NPR2* in ~2% of ~400 patients presenting with short stature [22]. The degree of short stature in these patients ranged from approximately -2 to -4 standard deviations.

As expected, mutations in *NPR2* causing short stature have been shown to lead to decreased levels of cGMP generation in cells transfected with mutant NPR-B receptors and stimulated with CNP [21]. The decreased activation of the CNP/NPR-B pathway leads to increased activation of FGFR3 signaling which should result in increased ERK1/2 activity and consequently decreased growth. As noted in the Clinical Hold letter, patients with homozygous mutations in *NPR2* are expected to be resistant to vosoritide therapy as neither copy of the NPR-B receptor, the target of vosoritide, is functional. However, patients who are heterozygous for *NPR2* mutations have one functional copy of *NPR2*, and it is plausible that vosoritide can increase cGMP generation through this functional receptor. A recent manuscript by Estrada et al. provides evidence supporting this notion [23]. In this manuscript, the authors first took a population genetics approach looking at several rare *NPR2* variants found in a large population-based dataset. They created an in vitro model of each variant by transfecting a plasmid encoding the mutant receptor into HEK293 cells. They were able to show that the different variants had a range of effects on cGMP generation. They then grouped the variants into categories based on cGMP generation, essentially categorizing the *NPR2* variants into buckets of severity. Once this was done, they compared the individual patient's height data with the severity of their *NPR2* mutation and found a strong statistical correlation between the two. This analysis demonstrates that there is a range of *NPR2* function in the population which correlates with height outcomes and suggests that if vosoritide can modulate the degree of signaling through NPR-B, it will lead to increased height.

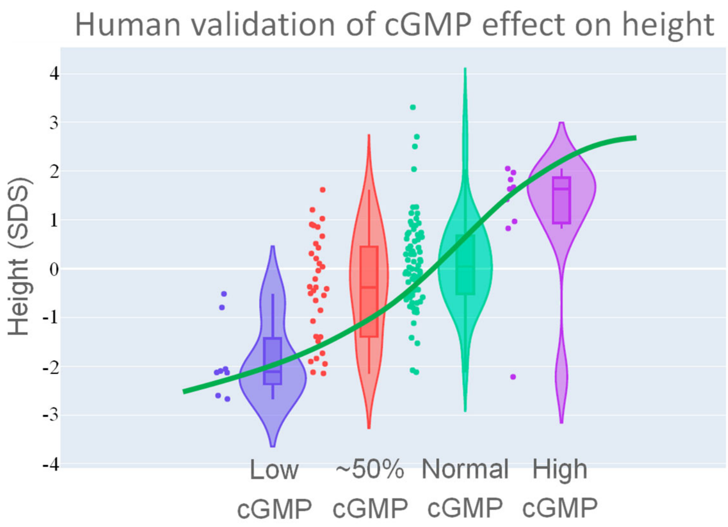

Figure taken from Reference [23]

As a next step, Estrada et al. [23] used genome editing to create rat chondrosarcoma cell lines in which *NPR2* was either completely knocked out or was haploinsufficient (i.e. mimicking a heterozygous loss-of-function mutation). They then performed a dose response experiment in which they added CNP and measured the cGMP generation. As expected, the homozygous knock out lines had absolutely no response to CNP. However, the heterozygous knock outs were able to respond to CNP, and at a concentration $\geq$0.163nM were able to generate a level of cGMP sufficient to activate downstream

protein kinase G signaling. Taken together, this data supports the notion that vosoritide will be able to increase signaling via the CNP/NPR-B pathway in patients with <u>heterozygous</u> *NPR2* mutations resulting in an ultimate increase in height.

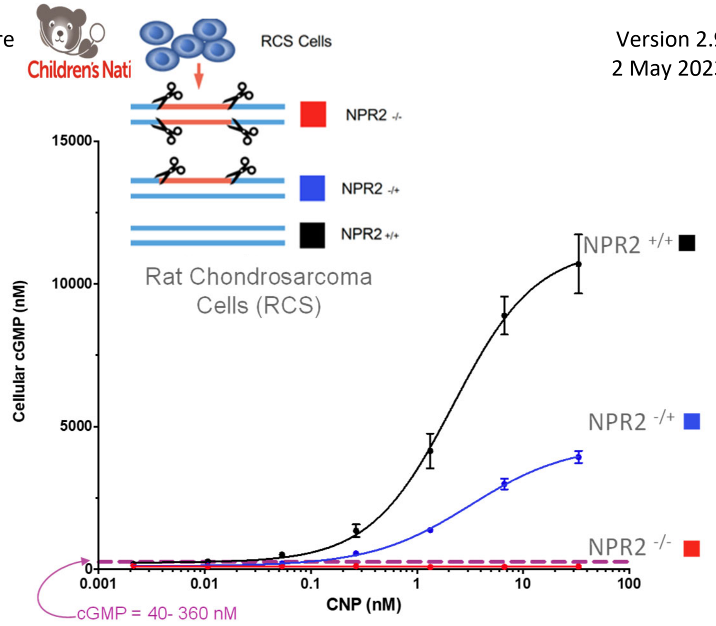

Figure taken from Reference [23]

<u>Rasopathy patients (including Noonan syndrome, Costello syndrome, Cardiofaciocutaneous syndrome, Neurofibromatosis Type 1)</u>

The Rasopathies are a group of disorders characterized by increased signaling through RAS/MAPK pathway. Noonan syndrome is the most common Rasopathy followed by Neurofibromatosis type 1 (NF1). This category also includes a number of extremely rare disorders such as Costello syndrome and Cardiofaciocutaneous syndrome. As demonstrated in the figure [24], this pathway leads to downstream activation of the RAF/MEK/ERK pathway. Short stature to varying degrees is a characteristic feature of the Rasopathies. CNP signaling intersects with this pathway by inhibiting RAF and leading to decreased MEK and ERK activation.

There are a number of lines of evidence that support the idea that vosoritide would be beneficial in patients with Rasopathies. In their *Endocrine Reviews* article [24], Tajan et al. extensively review the various genetic etiologies of Rasopathies and summarize the multiple animal models showing that mutations in these genes lead to increased activation of the RAS/MAPK pathway. In this article, they state “Coherent with the positive role of SHP2 on the RAS/MAPK pathway, functional analyses have shown that activating, NS-associated, SHP2 mutations hyperactivate ERK1/2. This effect has been repeatedly reported in different cell types in vitro, in the basal state as well as under stimulation by several agonists, and in different tissues/organs in animal models.” SHP2 is the protein encoded by *PTPN11*, the most common cause of Noonan syndrome.

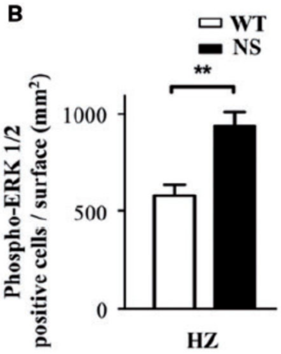

A mouse model of a *PTPN11* mutation causing Noonan syndrome has been shown to have short stature with decreased growth plate length and shortening of the hypertrophic zone [25]. Chondrocytes taken from these mice had increased phosphorylation of ERK1/2 (see

figure). In a second mouse model of Noonan syndrome due to a RAF1 mutation, Wu et al. found that these mice also have short stature and that MEK and ERK signaling was increased [26]. They then proceeded to give these mice a MEK inhibitor. Their results showed that "the body length of L613V/+ mice began to catch up with WT mice after 1 week of treatment, and by 2 weeks, PD0325901-treated L613V/+ mice were the same length as untreated WT mice [26]." MEK inhibition is downstream in the signaling pathway of RAF (see figure on prior page) which is the site where the CNP pathway intersects with the RAS/MAPK pathway. Thus, vosoritide is expected to be beneficial in this scenario.

As further evidence to support the use of vosoritide in Rasopathy patients, there are two highly relevant mouse studies in which CNP has been demonstrated to be an effective therapy in Rasopathy models. First, Ono et al. created mice deficient in *Nf1* in type II collagen producing cells [27]. These mice demonstrated constitutive ERK1/2 activation and decreased chondrocyte proliferation and maturation similar to what is seen in achondroplasia. They then performed daily injections of CNP in these mice which led to decreased ERK phosphorylation and corrected the short stature. Second, Inoue et al. created a mouse model of Cardiofaciocutaneous syndrome due to a specific *Braf* mutation (p.Q241R) [28]. These mice have decreased body length and reduced growth plate width with smaller proliferative and hypertrophic zones compared to wild type. Hypertrophic chondrocytes showed increased ERK activation. CNP administration led to increases in body length. Taken together, the in vitro and in vivo data suggest that the growth pathology seen in Rasopathy patients is at least partially due to increased signaling via ERK and that administration of a CNP analog offers the prospect of improved growth.

<u>Patients with SHOX deficiency</u>

SHOX is another gene implicated at the growth plate presenting with short stature. It encodes a transcription factor expressed throughout the growth plate with many functions including influencing the NPR-B and FGFR3 pathways. *SHOX* mutations have been estimated to account for approximately 2-10% of patients with idiopathic short stature [29,30]. The wide range of mechanistic effects of SHOX were extensively reviewed by Marchini et al. [30] and are summarized in the accompanying figure. Most relevant to the current protocol, SHOX has been shown to be a repressor of *FGFR3* transcription. Decker et al. used chromatin immunoprecipitation to demonstrate that SHOX directly binds to multiple upstream regulatory elements around *FGFR3* [31]. They then employed an in vitro model system for chondrogenesis and endochondral ossification using micromass cultures of chicken mesenchymal cells. Injecting a construct that expressed SHOX led to significant decreases in *Fgfr3* expression [31]. Thus, there is a clear link in the pathophysiology of SHOX deficiency and the FGFR3 pathway. SHOX deficiency leads to increased FGFR3 signaling, similar to what is seen in achondroplasia, making vosoritide a logical treatment choice. Additionally, there is some evidence to support that SHOX has direct interactions with CNP/NPR2 as well [30].

<u>Patients with heterozygous defects in *ACAN* (the gene that encodes Aggrecan)</u>

Aggrecan is a proteoglycan found in the extracellular matrix of growth plate cartilage. Individuals with heterozygous defects in Aggrecan present with dominantly inherited short stature often with advanced bone ages and premature growth cessation [32,33]. A chick model of Aggrecan deficiency demonstrated the absence of extracellular matrix between chondrocytes as well as smaller hypertrophic chondrocytes with premature differentiation of the chondrocytes into the hypertrophic phenotype [34]. Interestingly, this model showed evidence of FGFR3 dysregulation with increased levels of phosphorylated ERK in the mutant cartilage (see Figure) [34]. In addition to the inhibitory effect on the ERK pathway detailed above, CNP has also been shown to normalize the decreased extracellular space seen in an achondroplasia mouse model and to increase glycosaminoglycan synthesis in those mice [35]. In a study examining the effects of FGF2 on rat chondrosarcoma cells, CNP treatment was also able to rescue the loss of extracellular matrix seen after FGF2 administration [36]. Through detailed mechanistic studies, the authors found that CNP inhibited matrix metalloproteinases, proteins that degrade the extracellular matrix components, in addition to stimulating extracellular matrix production. They noted that this effect on matrix production was independent of endogenous FGF2 signaling [36]. Based on these studies, we conclude that vosoritide therapy may be beneficial in patients with Aggrecan deficiency as it will both inhibit the increased ERK phosphorylation seen in Aggrecan deficiency as well as induce increased production of extracellular matrix through FGF2 independent methods.

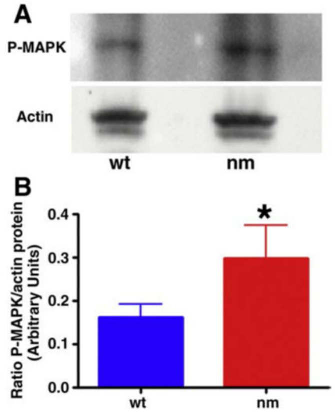

Fig. 10. Phosphorylated MAPK is increased in E6 nm cartilage. (A) Immunoblotting analysis showing levels of phosphorylated MAPK (Erk), and β-actin as a loading control, in E6 wt and nm cartilage lysates. (B) Relative levels of phosphorylated MAPK to β-actin were quantified in three independent experiments. Data was evaluated for statistical significance using the Student's *t*-test. $^{*}p<0.04$.

In sum, each of these six defined genetic etiologies has a direct mechanistic interaction with the CNP/FGFR3 pathways and the available scientific evidence supports a prospect of benefit of vosoritide treatment for patients with genetic defects in these pathways.

As noted above, mutations in CNP and its receptor *NPR2* lead to significant short stature. On the flip side, a number of patients have been identified with over activation of this signaling pathway. There are 4 patients described who had tall stature due to translocations leading to overexpression of CNP [37–39]. All of these patients had significant tall stature with varying degrees of skeletal changes. Additionally, three manuscripts describe patients with rare activating mutations of *NPR2* who present with a similar phenotype of tall stature and mild skeletal changes [40–42]. Finally, transgenic mouse models which overexpress CNP have increased length due to increased proliferation of chondrocytes within the growth plate [43]. All of this data taken together suggest that increased signaling through the CNP/NPR2 pathway enhances linear growth via a direct effect at the growth plate chondrocyte. These patients also did not demonstrate signs of non-skeletal morbidity suggesting that increased signaling through this

pathway should be relatively safe without significant off-target effects. This human genetic data in addition to the clinical trial results from BioMarin's achondroplasia studies support our hypothesis that vosoritide could lead to increased stature in a range of conditions affecting the growth plate.

We will perform a single arm, open-label, Phase I/II study of vosoritide in patients with the selected genetic causes of short stature identified above. This will be a single center study performed at Children's National Hospital under the direction of Dr. Andrew Dauber, Chief of Endocrinology. We will enroll patients who meet our inclusion criteria and then follow them for a 6 month observation period in order to obtain baseline growth velocity data, safety profile, and quality of life assessment. We will then treat them with vosoritide SC at the 15 µg/kg/day dose. This dose was selected because it was equally efficacious to the 30 µg/kg/day dose in the Phase II trial for achondroplasia and had a good safety profile. While it is possible that patients who meet this study's inclusion criteria could have a more robust response to a higher dose such as the 30 µg/kg dose, a more conservative approach was chosen for this initial exploratory proof of principle study. The subjects will then be dosed for 1 year. Subjects with a positive response to therapy will be given the option to continue in the extension phase of the study during which they will continue to receive vosoritide until growth cessation.

2.3 RISK/BENEFIT ASSESSMENT

2.3.1 KNOWN POTENTIAL RISKS

Vosoritide is an investigational agent and therefore all possible risks are not yet known. Vosoritide has thus far been tested in animals, adult male healthy volunteers and children with achondroplasia. Based on the current experience with vosoritide, the known risks include the following:

1. Local injection site reactions – Overall, injection site reactions were the most common adverse event noted in trials to date with ~85% of subjects having some form of injection site reaction. These have all been minor and have self-resolved with no subjects requiring discontinuation of vosoritide due to a site reaction.
2. Hypotension and reflex tachycardia – Vosoritide could theoretically lead to hypotension. In studies to date, ~45% of subjects had hypotension documented on vital sign measurement. All events of hypotension were non-serious and self-resolved without intervention. All except one were asymptomatic. According to the Investigator's Brochure [9], "The one symptomatic event occurred in subject enrolled in Cohort 1 (2.5 µg/kg/day). The subject experienced an event reported as "dizziness secondary to symptomatic hypotension" (preferred term: hypotension) on study Day 14. The event began 56 minutes after the subject received study drug, and resolved without medical intervention in 5 minutes. The event occurred at home, and therefore no BP measurements were available during the episode of dizziness. Additionally, 3 subjects receiving BMN 111 at 15 µg/kg were reported to have transient decrease in BP in the context of other reported adverse events as the underlying diagnosis. One subject experienced 2 events of syncope, with drop in BP and heart rate, and both were attributed to blood draws. Another subject experienced 1 event of presyncope with drop in BP associated with micturition. Both subjects remained in the study and continued to tolerate study drug without recurrence of symptoms. Another subject reported an event of presyncope (without drop in BP) related to prolonged standing for anthropometric assessments. As of the data cutoff, one subject (3.3%) in 111-205 has reported an event of hypotension. The subject had an event of symptomatic

hypotension (Grade 2) associated with dizziness on Day 281, as well as 2 events of asymptomatic hypotension on Days 379 and 550. The symptomatic event began 30 minutes after the subject received study drug, with a blood pressure of 88/54 mmHg. The event resolved without medical intervention."

3. Allergic reaction – To date, there have been no serious hypersensitivity reactions to vosoritide but this is always a theoretical risk with an investigational drug.
4. Excessive growth – It is theoretically possible that research subjects could have a very robust response to vosoritide treatment. This could lead to excessive growth and potentially exacerbation of underlying disproportionality. This will be monitored at study visits and is unlikely to occur.
5. Risks from phlebotomy and injections – With any blood draw, IV placement, or subcutaneous injection, there exists a small risk of local bruising, erythema, infection, and syncope.
6. Risks from genetic testing – With any genome wide genetic testing, there is a potential risk that someone could identify the subject from their genetic information and use this information to discriminate in some way. These risks may also affect members of the subject's family. This risk will be mitigated as all of the genetic information will be kept confidential as part of the research record.
7. Risks from X-rays - Every person is exposed on a daily basis to a certain amount of background radiation originating from soil, rocks, outer space and within the body itself. The amount of radiation for imaging studies varies based on the size of the patient, with children and smaller adolescents being exposed to relatively larger doses of radiation than adults. Subjects in this study will be exposed to an acceptable amount of radiation similar to what would occur in routine clinical care.

2.3.2 KNOWN POTENTIAL BENEFITS

Patients may benefit from treatment with vosoritide through improved growth and increased growth velocity. This could affect their ultimate final adult height. Improvements in growth through treatment with growth hormone have been associated with improved quality of life measures in children with short stature [44]. In the patients with achondroplasia, there was a mean increase of 1.03 SDS in height from baseline after 42 months of intervention using the 15 µg/kg dose [2]. It is quite possible that patients with other causes of short stature will have an even more favorable response as they will have less severe perturbations in growth plate physiology.

2.3.3 ASSESSMENT OF POTENTIAL RISKS AND BENEFITS

The overall safety profile for vosoritide has been extremely reassuring to date with minimal significant adverse events. Patients with other genetic causes of short stature lack highly beneficial therapies and some are quite motivated to pursue therapy to improve their stature. We are intentionally starting with a conservative dose of 15 µg/kg/day dose because it was equally efficacious to the 30 µg/kg/day dose in the Phase II trial for achondroplasia [2]. While it is possible that patients with the selected genetic causes of short stature included in this study could have a more robust response to a higher dose such as the 30 µg/kg dose, we decided to start with the lowest previous effective dose as this is an initial exploratory proof of principle study. We believe that the potential benefits of this therapy outweigh the risks in this study population.

3 OBJECTIVES AND ENDPOINTS

OBJECTIVES	ENDPOINTS	JUSTIFICATION FOR ENDPOINTS
Primary		
The primary objectives of the study are as follows:<br>• To evaluate the safety and tolerability of daily subcutaneous (SC) injections of vosoritide administered for 12 months in patients with selected genetic causes of short stature<br>• To evaluate the change from baseline in age-sex standardized annualized growth velocity after 12 months of daily SC injections of vosoritide in patients with selected genetic causes of short stature<br>• To evaluate the change from baseline in age-sex standardized height standard deviation score (SDS) after 12 months of daily SC injections of vosoritide in patients with selected genetic causes of short stature	The primary study endpoints include:<br>• Incidence of adverse events per subject with particular attention to Grade 3 and 4 adverse events as well as to rates of hypotension<br>• Change from baseline in age-sex standardized annualized growth velocity after 12 months of treatment<br>• Change from baseline in age-sex standardized height SDS after 12 months of treatment	The rates of adverse events will describe the safety profile of vosoritide in this study population.<br><br>Change in annualized growth velocity and height SDS are standard measures of efficacy of growth promoting agents. They are highly correlated but as height velocity differs by age and gender, the height SDS takes these factors into account.
Secondary		
The secondary objectives of the study are as follows:<br>• To evaluate changes in body proportions from baseline after 12 months of daily SC injections of vosoritide<br>• To evaluate changes from baseline in bone age/chronological age after 12 months of daily SC injections of vosoritide	The secondary study endpoints include:<br>• Change from baseline in seated height ratio after 12 months of treatment<br>• Change from baseline in arm span minus standing height after 12 months of treatment<br>• Change from baseline in bone age/chronological age after 12 months of treatment	Seated height ratio and arm span minus standing height are standard measures of disproportionality. It is possible that vosoritide good affect axial versus appendicular growth in different ways and thus we want to assess its effect on proportionality.

OBJECTIVES	ENDPOINTS	JUSTIFICATION FOR ENDPOINTS
		Bone age is used to predict remaining height potential. As vosoritide has the potential to advance bone age, the measurement of the ratio of bone age to chronological age is a standard way of evaluating if the growth promoting agent is leading to premature bone age advancement which may limit growth potential.
Tertiary/Exploratory		
The exploratory objectives of the study are as follows:<br>• To evaluate the pharmacokinetic (PK) profile of vosoritide in patients with selected genetic causes of short stature<br>• To evaluate changes from baseline in bone mineral density (BMD) after 12 months of daily SC injections of vosoritide<br>• To evaluate changes from baseline in vosoritide activity biomarkers and bone/collagen biomarkers after 12 months of daily SC injections of vosoritide<br>• To evaluate immunogenicity and assess impact on safety and efficacy measures after 12 months of daily SC injections of vosoritide	The exploratory study endpoints include:<br>• Standard PK parameters including Cmax, Tmax, half-life, etc…<br>• Change from baseline in total body BMD Z-score adjusted for height.<br>• Change from baseline in lumbar spine BMD Z-score adjusted for height.<br>• Change from baseline in cGMP and serum collagen X markers.<br>• Rates of vosoritide antibody development.<br>• Change from baseline in QoLISSY scale	We assume that this patient population will have similar PK parameters to those previously described in patients with achondroplasia but want to confirm that this assumption is correct.<br><br>As CNP affects bone growth parameters, we would like to explore the effects on bone mineral density as a safety measure.<br><br>cGMP and serum collagen X markers are pharmacodynamics measures of vosoritide efficacy. We want to explore the PK/PD relationship in this patient population and compare to that seen

OBJECTIVES	ENDPOINTS	JUSTIFICATION FOR ENDPOINTS
• To explore the relationship between genetic etiology and safety and efficacy<br>• To evaluate changes from baseline in quality of life using the QoLISSY scale after 12 months of daily SC injections of vosoritide		in achondroplasia patients.<br><br>Vosoritide antibody assessment is a standard safety metric.<br><br>QoLISSY is a validated scale for quality of life measurement in children with short stature.

During the extension study, all of the primary, secondary and exploratory aims and endpoints will remain the same, but the timeframe will be extended from 12 months until growth cessation or subject withdrawal from the study.

4 STUDY DESIGN

4.1 OVERALL DESIGN

We hypothesize that patients with selected genetic causes of short stature will benefit from treatment with vosoritide, a CNP analog which directly targets the growth plate. The selected genetic causes included in this study all have in vitro and in vivo evidence to suggest a prospect of benefit from vosoritide treatment.

This is a pediatric open-label, Phase I/II, single arm study of up to 75 subjects with selected genetic causes of short stature. This is a single site study being performed at Children's National Hospital. Subjects who are 3 to 10 years old inclusive for males and 9 years old inclusive for females who meet the clinical inclusion criteria defined below and meet all eligibility requirements will participate. After enrollment, subjects will be followed for a 6-month observation only period to establish a baseline height velocity, safety profile, and quality of life assessment. Vosoritide will then be administered daily via subcutaneous injection at a dose of 15 µg/kg/day for 12 months. Subjects with a positive response to therapy will be given the option to continue in the extension phase of the study during which they will continue to receive vosoritide until growth cessation.

4.2 SCIENTIFIC RATIONALE FOR STUDY DESIGN

In this study, we are focusing on six specific genetic subgroups causing short stature. These six groups all share a common final pathway of growth inhibition via increased activation of ERK1/2 signaling in the chondrocyte (the pathway targeted by vosoritide). These groups are also each relatively rare and within each group there is significant heterogeneity in the severity of short stature. Therefore, we think it is reasonable to combine the six groups into a single study cohort. To control for the interindividual variability, each subject will serve as his or her own control via the baseline 6 month observation only period. We propose to include up to 75 subjects in order to have at least 10 subjects in each subgroup. As the response to vosoritide therapy in this group is unknown, no formal power calculations are

planned. Rather, this is an exploratory study in a rare disease population, and we seek to obtain initial data which will influence future study planning. Additionally, at this time, it is not feasible to recruit a large enough cohort of any of the specific genetic causes to make this a meaningful study. It is difficult to predict the anticipated distribution of patients between each subgroup. In addition to the whole cohort analysis, exploratory analyses will be performed based on underlying genetic etiology. In the future, a randomized controlled study will be required in order to definitively prove efficacy. That is not the goal of the current study as this is a proof or principle study to assess whether subjects meeting these inclusion criteria have the potential to respond to vosoritide therapy.

4.3 JUSTIFICATION FOR DOSE

The 15 µg/kg/day dose was chosen based on the results from the Phase II study in achondroplasia in which this dose was equally efficacious to the 30 µg/kg/day dose in the Phase II trial for achondroplasia [2]. While it is possible that patients meeting our study inclusion criteria could respond even better to a higher dose such as the 30 µg/kg dose, we decided to start with the lowest previous effective dose as this is an initial exploratory proof of principle study.

4.4 END OF STUDY DEFINITION

A participant is considered to have completed the study if he or she has completed all phases of the study including the last visit or the last scheduled procedure shown in the Schedule of Activities (SoA), Section 1.3.

The end of the study is defined as completion of the last visit or procedure shown in the SoA in the trial globally.

5 STUDY POPULATION

5.1 INCLUSION CRITERIA

Inclusion and exclusion criteria will be pre-screened based on information provided by the referring clinician. All inclusion and exclusion criteria will then be verified at the screening visit and official screening laboratory studies will be performed as part of that visit. There will be no birth weight or length inclusion or exclusion criteria.

In order to be eligible to participate in this study, an individual must meet all of the following criteria:

1. Parent(s) or guardian(s) are willing and able to provide written, signed informed consent after the nature of the study has been explained and prior to performance of any research-related procedure. Also, subjects under the age of 18 are willing and able to provide assent (if required) after the nature of the study has been explained and prior to performance of any research-related procedure.
2. Stated willingness to comply with all study procedures and availability for the duration of the study
3. Age ≥3 years 0 days AND ≤10 years 364 days for males, ≤9 years 364 days for females
4. Pre-pubertal defined as Tanner Stage 1 breasts in females and testicular volumes ≤3 cc in males. This must be confirmed at the Day 1 visit prior to initiation of vosoritide.
5. Patient height ≤-2.25 SDS. All height SDS values are calculated using the CDC growth charts/data tables [45].

6. Patients with pathogenic or likely pathogenic variants in genes known to cause the specific genetic subgroups of short stature listed below are eligible for inclusion in the study. Pathogenicity of variants will be classified as per the American College of Medical Genetics criteria with the exception of *ACAN* mutations as detailed below [46]. Documentation of the presence of the variant must be obtained using a lab results report from a CLIA certified laboratory. Classification of the variant's pathogenicity status will be performed by the Children's National study team.
    A. CNP deficiency due to mutations in *NPPC* – Subjects with heterozygous or homozygous defects in *NPPC* are eligible.
    B. Hypochondroplasia – Subjects with heterozygous variants in *FGFR3* gene associated with hypochondroplasia are eligible. Subjects with variants in *FGFR3* known to cause achondroplasia or thanatophoric dysplasia or SADDAN syndrome will be excluded.
    C. Patients with heterozygous defects in *NPR2* are eligible. Patients with homozygous defects in *NPR2* will be excluded.
    D. Rasopathy patients (including Noonan syndrome, Costello syndrome, Cardiofaciocutaneous syndrome, Neurofibromatosis Type 1) – This include patients with heterozygous variants in the following genes:
        i. BRAF
        ii. CBL
        iii. HRAS
        iv. KRAS
        v. LZTR1
        vi. MAP2K1
        vii. MAP2K2
        viii. MRAS
        ix. NF1
        x. NRAS
        xi. PPP1CB
        xii. PTPN11
        xiii. RAF1
        xiv. RRAS
        xv. RIT1
        xvi. SHOC2
        xvii. SOS1
        xviii. SOS2
    E. Patients with SHOX deficiency – Patients with either heterozygous, compound heterozygous or homozygous defects in *SHOX* including patients with heterozygous or homozygous deletions of the *SHOX* regulatory region known to cause SHOX deficiency.
    F. Patients with heterozygous defects in *ACAN* - Patients must be heterozygous for a mutation in the *ACAN* gene. As there are no validated *in vitro* assays that reliably assess an individual variant's effect on aggrecan function, for the purpose of this clinical trial a mutation in *ACAN* will be defined as:

a. A heterozygous deletion of the entire gene or of ≥1 complete exons of the gene
b. Any truncating mutation including frameshift, nonsense, splice site mutations within 2 bases of the exon/intron boundary, and start loss variants
c. Any missense mutation which meets all of the following criteria:
    i. It has an aggregate minor allele frequency less than $1X10^{-5}$ based on the gnomAD data (gnomad.broadinstitute.org)
    ii. It is predicted to be damaging by BOTH Polyphen2 and SIFT
    iii. It segregates with the short stature phenotype in available family members or is a *de novo* mutation
d. In-frame insertions or deletions of >1 amino acid
e. In-frame insertions or deletions of 1 amino acid must meet the same criteria as missense mutations. For the prediction programs, Alanine will be substituted for the deleted amino acid.

7. Absence of growth hormone deficiency defined as an IGF-1 level above the lower limit of the normal range of the assay. The IGF-1 may be repeated during the 6 month observation period and prior to the Day 1 visit. If this repeat IGF-1 is above the lower limit of the normal range of the assay, then the subject is deemed eligible. If a patient has an IGF-1 level below the lower limit of the normal range of the assay, two growth hormone stimulation tests must be performed using the routine local protocols. Patients with a peak growth hormone level ≥7 ng/ml will be considered growth hormone sufficient and will be eligible for inclusion as per the Growth Hormone Research Society International consensus [47]. If indicated based on IGF-1 level from the referring clinician, the growth hormone stimulation test must be done as part of routine clinical care prior to enrollment. The rationale for using an IGF-1 below the normal range as the cut-off for further evaluation for growth hormone deficiency is that in patients with a clear genetic explanation for their short stature, an IGF-1 level anywhere within the normal range would be considered reassuring and would not lead to a growth hormone stimulation test in a routine clinical setting.
8. The subject and their guardian must speak one of the 11 languages for which the QoLISSY survey (a quality of life survey for short stature) is available. These include: English, Spanish, German, Russian, Swedish, Flemish, Italian, Turkish, French, Japanese, and Ukrainian.

Extension Phase Inclusion Criteria:

1. Subject completed all visits for the initial 18 months of the study.
2. Parent(s) or guardian(s) are willing and able to provide written, signed informed consent for the extension phase after the nature of the study has been explained and prior to performance of any research-related procedure. Also, subjects under the age of 18 are willing and able to provide assent (if required) after the nature of the study has been explained and prior to performance of any research-related procedure.
3. Subject must have a positive response to the first year of vosoritide therapy which is defined as:
    - For subjects with hypochondroplasia or *ACAN* mutations – A positive response is defined as an increase in annualized growth velocity of 1.6 cm/year when comparing 6-month observation period to initial 12 months of vosoritide treatment. This criterion is based on the adjusted mean response of patients with achondroplasia in the Phase III trial of

vosoritide which is a clinically significant response[48]. *ACAN* patients can also have disproportion and are included in this category.

- For subjects with Rasopathies, *SHOX* mutations, *NPR2* mutations or CNP deficiency - A positive response is defined as an increase in annualized growth velocity of 3 cm/year when comparing 6-month observation period to initial 12 months of vosoritide treatment. This higher response criterion is due to growth hormone being approved for SHOX deficiency, Noonan syndrome, and for idiopathic short stature which could encompass *NPR2* mutations and CNP deficiency. In these types of patients, an increase in annualized growth velocity of 3 cm/year is more typical for patients treated with growth hormone, and thus, we want to ensure that patients treated with vosoritide have a similarly favorable response. However, if a subject has previously been treated with growth hormone and it was discontinued due to a poor response, if the increase in annualized growth velocity on vosoritide is greater than it was for growth hormone but still less than 3 cm/year, the subject can continue on vosoritide in the extension phase.

4. There are no inclusion criteria based on height or pubertal status for the extension phase of the protocol. Subjects may continue into the extension phase regardless of their height or pubertal status as the 12 Month Visit.

5.2 EXCLUSION CRITERIA

An individual who meets any of the following criteria will be excluded from participation in this study:

1. Growth plate fusion – Defined as a bone age via the Greulich and Pyle method of 13 years in females and 15 years in males. These patients have limited remaining growth potential.
2. Concomitant treatment with growth hormone or recombinant IGF-1. Patients may have been previously treated with growth hormone or IGF-1 therapy. If the patient is currently on one of these therapies, they will be required to discontinue treatment in order to begin the baseline observation period for this trial. That decision will be deferred to their treating clinical endocrinologists in conjunction with the patient's guardians. We anticipate that only patients who are having a poor response to their therapy will be interested in enrolling in the current study as there is no rationale for a patient who is receiving growth hormone therapy and having a positive response to enroll in the current study.
3. Prior treatment with a GnRH analog, aromatase inhibitor or oxandrolone
4. History of any type of malignancy
5. Chronic medical condition known to affect growth including but not limited to:
    - A. Cystic fibrosis
    - B. Diabetes
    - C. Inflammatory Bowel Disease
    - D. Celiac Disease
    - E. Asthma requiring a daily inhaled steroid dose > 400 micrograms of inhaled budesonide per day or equivalent
    - F. Taking daily oral glucocorticoids for any reason
    - G. Note – ADHD treated with a stimulant and treated hypothyroidism with a normal TSH will NOT exclude the subject from participating in the trial.
    - H. Turner Syndrome or any other chromosomal aneuploidy

    I. Congenital heart disease which places the subject at increased risk of an adverse cardiac outcome in the setting of hypotension including but not limited to: hypertrophic cardiomyopathy, aortic stenosis with peak gradient >50mmHg, severe aortic regurgitation (defined as pressure half time >500ms by echocardiogram), coronary insufficiency, or any anatomy with a need for an afterload reducing agent. Any patient with baseline abnormalities on echocardiogram will be reviewed with a pediatric cardiologist for appropriateness for inclusion in the study.

6. Malnutrition – Defined as a BMI <5th percentile (CDC growth charts)
7. Any clinically significant abnormality on screening tests as determined by the principal investigator. Abnormal screening labs may be repeated during the 6 month observation period prior to Day 1. If they return to normal or non-clinically significant deviations per the PI's determination, the subject may proceed with the study.
8. Known or suspected allergy to trial medication, excipients, or related products
9. The receipt of any investigational drug within 90 days prior to this trial

Extension Phase Exclusion Criteria:
At the 12 Month Visit, the subject's bone age via the Greulich and Pyle method will not be greater than or equal to 13 years in females and 15 years in males. These patients have limited remaining growth potential.

None of the other exclusion criteria will be applied to the extension phase of the study as they would have been dealt with as adverse events during the trial.

5.3 LIFESTYLE CONSIDERATIONS

Not applicable

5.4 SCREEN FAILURES

Screen failures are defined as participants who consent to participate in the clinical trial but are not subsequently assigned to the study intervention or entered in the study. A minimal set of screen failure information is required to ensure transparent reporting of screen failure participants, to meet the Consolidated Standards of Reporting Trials (CONSORT) publishing requirements and to respond to queries from regulatory authorities. Minimal information includes demography, screen failure details, eligibility criteria, and any serious adverse event (SAE).

The most likely reasons for screen failures in this study are that the screening IGF-1 level done during the screening visit is below the normal range on our assay and a growth hormone stimulation test has not previously been performed. In those cases, the subjects will be referred back to their primary pediatric endocrinologist who can elect to perform a growth hormone stimulation test at their discretion. If the growth hormone stimulation test is performed and the subject meets study inclusion criteria after the results are available, the subject will then be deemed eligible for the study and will no longer be considered a screen failure. They will then continue with the protocol as scheduled.

5.5 STRATEGIES FOR RECRUITMENT AND RETENTION

Research subjects will be directly recruited at Children's National Hospital and will be referred to the study by pediatric endocrinologists and geneticists throughout the United States. Direct recruitment at Children's National will occur when members of the endocrinology or genetics division identify patients who meet the inclusion criteria. The study will be publicized throughout the United States in a number

of ways. First, the study will be listed on national websites including clinicaltrials.gov as well as the Pediatric Endocrine Society website. Second, an email will be sent to the pediatric endocrinology list serve describing the study and providing information about how to refer to the study. Third, Dr. Dauber (the PI) will publicize the study during presentations at national meetings and when he speaks at other institutions. Additionally, study brochures will be handed out at the annual Pediatric Endocrine Society meeting. Fourth, Dr. Dauber will send direct emails to pediatric endocrinologists and geneticists throughout the US informing them about the study and providing information about how to refer eligible patients. All subjects will be recruited from within the United States.

Once a subject has been referred by their pediatric endocrinologist, we will then arrange for a telephone call to review the study details and confirm that the patient meets the inclusion and exclusion criteria. Prior to the telephone call, a copy of the consent forms will be provided to the family. During the telephone call, we will review all study procedures and answer any questions from the subject and their guardian(s). If, after the telephone call, the subject and guardian are interested in enrolling in the study, we will make arrangements for the screening visit which will also serve as the start of the observation period.

As the inclusion criteria are quite straightforward, we anticipate a small number of screen failures and estimate that we will screen up to 100 subjects to enroll the cohort of up to 75 subjects.

To enhance study retention, a minimum of monthly phone calls are scheduled once the intervention begins. We anticipate that subjects enrolled in this study will be highly motivated to complete the study and do not anticipate a high dropout rate.

The study will cover all travel costs for the subject and 1 parent including flights or mileage reimbursement for those who choose to drive, 2 nights in a hotel, transportation costs to and from the airport and to and from the hospital, as well as up to $50 per person per day for meals. No additional incentives will be provided. We want to alleviate the financial burden of study participants but are not looking to incentivize subjects to participate in the study.

6 STUDY INTERVENTION

6.1 STUDY INTERVENTION(S) ADMINISTRATION

6.1.1 STUDY INTERVENTION DESCRIPTION

Vosoritide, also known as BMN 111 or modified recombinant human C-type natriuretic peptide (CNP), is a 39-amino-acid peptide analog that includes the 37 C-terminal amino acids of the human CNP53 sequence plus the addition of 2 amino acids (Pro-Gly) on the N-terminus. This structural modification conveys resistance to neutral endopeptidase (NEP) degradation, resulting in prolonged half-life (t1/2) in comparison to endogenous CNP. This increase in t1/2 allows once daily subcutaneous (SC) administration.

Vosoritide is currently an investigational drug and is not commercially available.

6.1.2 DOSING AND ADMINISTRATION

During the study, vosoritide will be administered as a single 15 µg/kg subcutaneous injection given daily at approximately the same time each day whenever possible. The time of day of administration will be up to the individual subject and their guardian. It will be given 7 days per

week. The same injection site should not be used 2 days in a row, and sites should be rotated. Doses may be administered in any of the common subcutaneous areas (upper arm, thigh, abdomen, buttocks). If a dose is missed and noticed within 12 hours of the missed dose, it should be given at that time. If it has been more than 12 hours since the time of the missed dose, that dose should be skipped and the regular dose should be given the following day.

Vosoritide will be given for an initial 12-month period followed by an extension phase until cessation of growth or subject withdrawal from the study or until vosoritide becomes commercially available for the subject's indication. Doses will be given in the clinic during the Day 1, Day 2, 6 Month and 12 Month Visits prior to pharmacokinetic sampling. At the 6 month visit, the subject will be weighed. The dose of medication will be recalculated using the same 15 µg/kg dose but adjusted for the new weight. There will be no other dose increases in the protocol. After consultation with the DSMB, the dose may be reduced by 20% if the subject meets the individual stopping criteria on one occasion as detailed in **Section 7.1** below.

Subjects with a good response at 12 months will be offered participation in the extension phase of the study. A good response is defined in the inclusion criteria above. Subjects in the extension phase will continue to be seen in-person every 6 months at Children's National with telephone visits at the 3-month time point mid-way between in-person visits.

6.2 PREPARATION/HANDLING/STORAGE/ACCOUNTABILITY

6.2.1 ACQUISITION AND ACCOUNTABILITY

Vosoritide will be supplied by BioMarin to the Investigational Pharmacy at Children's National Hospital in sterile, single-dose, Type I glass vials with coated stopper and flip-off aluminum cap. Vosoritide drug product is supplied as a lyophilized, preservative-free, white-to-yellow powder for reconstitution with pre-filled sterile water for injection syringes. BioMarin will provide all additional necessary supplies for drug administration. The study team will work closely with the investigational pharmacy to monitor drug supply. Details of needed supplies and protocol for requesting additional drug from BioMarin will be detailed in a Pharmacy Manual prior to initiation of the study.

The investigational pharmacy will distribute 3 month supplies of medication, pre-filled sterile water for injection syringes, syringes and all necessary supplies to each study subject via the investigator at the baseline and 6 month study visits and at all in-person extension study visits. An additional 3 month supply will be shipped to the subjects at months 3 and 9 of the study and at the necessary intervals during the extension phase. Drug and supplies will be packed and shipped overnight from the IDS with the assistance of the study team via FedEx. Study drug kits and supplies do not require refrigeration during transportation from the IDS to the subject's home, but must be refrigerated when stored at the subject's home. Additional details regarding all these aspects will be provided in the Pharmacy Manual. The study team will contact the family the day after shipping to ensure that undamaged product and supplies were received. At the 6 month and 12 month visit and all in-person extension study visits, the subjects will return any used (empty) and unused drug product vials, unused sterile water for injection syringes, and unused supplies to the investigator which will then be counted as a measure of drug administration compliance. At the 12 month study visit or at the final study visit in the extension study, used (empty) and unused study drug, unused pre-filled sterile water for injection syringes, and supplies will be returned to the investigational pharmacy for disposal. At the conclusion of the entire study,

unused drug will either be returned to BioMarin or destroyed as per standard operating procedure of the investigational pharmacy.

6.2.2 FORMULATION, APPEARANCE, PACKAGING, AND LABELING

Vosoritide is cloned into the pJexpress401 vector, expressed in E. coli and then purified. The drug substance is a modified CNP peptide that retains wild-type activity and specificity. The modified CNP sequence is:

PGQEHPNARKYKGANKKGLSKGCFGLKLDRIGSMSGLGC

The amino acid sequence is an analogue of the naturally occurring tissue-expressed form of C-type natriuretic peptide (CNP-53). Vosoritide is a recombinant 39 amino acid peptide that includes the 37 C-terminal amino acids of the human CNP-53 sequence, and is engineered to include two additional amino acids (Pro-Gly) on the N-terminus, which renders the peptide more resistant to degradation. It is a cyclic peptide formed by an intramolecular disulfide bond. The molecular weight of the purified product is 4.1 kDa.

The clinical drug product will be supplied in sterile, single-dose, Type I glass vials with coated stopper and flip-off aluminum cap. The vial will have a label stating “Caution: New Drug--Limited by Federal (or United States) law to investigational use." Vosoritide drug product is supplied as a lyophilized, preservative-free, white-to-yellow powder for reconstitution with pre-filled sterile water for injection (WFI) syringes. The reconstituted solution is colorless to yellow and contains 0.8 mg/mL to 2 mg/mL of vosoritide, as well as citric acid, sodium citrate, trehalose, mannitol, methionine, polysorbate 80, and sterile WFI. The target pH of the reconstituted solution is 5.5. Sterile WFI will be supplied in a pre-filled diluent syringe. All reconstitution and dose preparation steps will be performed as indicated in the vosoritide Injection Guide and Injection video. The subjects and their guardians will be trained in proper techniques for drug reconstitution and administration during the Day 1 Visit. Practice supplies will be provided along with educational materials. The subjects or their guardian will be required to demonstrate proper technique during the educational session. At the 6 month visit, we will review administration technique.

6.2.3 PRODUCT STORAGE AND STABILITY

Lyophilized vosoritide should be stored in a restricted area at refrigerated temperature (2°C-8°C; 36°F–46°F). All reconstituted vosoritide must be administered as a SC injection within 3 hours of reconstitution. Drug will be provided in a secure lock box which also contains a temperature monitor. Details of drug storage and temperature monitoring will be provided in the Pharmacy Manual. A drug administration log will be provided to the participants on which they will record the temperature information as well as the timing of reconstitution, timing of drug administration and administration site.

6.2.4 PREPARATION

Details of study drug preparation are available in the separate pharmacy guide. All reconstitution and dose preparation steps will be performed as indicated in the vosoritide Injection Guide and accompanying video which will be provided to study participants. Complete details of all study drug handling will be in the study SOP. In brief, vosoritide must remain at 2-4 degrees Celsius at all times. The study drug will be shipped by BioMarin to the investigational pharmacy where it will be kept in cold storage with appropriate monitoring of temperature. A 3 month supply will be dispensed to the family at Visit Day 2 in a special cooler which will maintain the appropriate temperature during transport to the family’s home. Each day, the family will remove a single vial from the study drug box. After allowing the

vial to sit at room temperature for 30 minutes, the parent/guardian will add the contents of the diluent syringe into the vial with vosoritide and then swirl the vial until all drug is dissolved. They will then let the vial sit for 5 minutes before drawing the correct dose of medication into an injection syringe.

6.3 MEASURES TO MINIMIZE BIAS: RANDOMIZATION AND BLINDING

Not applicable. This is an open label proof of concept study. There is no planned randomization or blinding.

6.4 STUDY INTERVENTION COMPLIANCE

Adherence to the protocol will be monitored in a number of ways. First, subjects will be given a drug log in which they will record each day's administration including timing of drug reconstitution, time of administration, temperature confirmation, and site of administration. Second, we will have periodic telephone calls with the subjects during which medication adherence will be reviewed (See Schedule of Activities, **Section 1.3**). Third, subjects will be asked to bring all remaining medication vials to the 6 and 12 month study visits and all extension phase in-person visits. Vial counts will be performed as a measure of compliance.

6.5 CONCOMITANT THERAPY

For this protocol, a prescription medication is defined as a medication that can be prescribed only by a properly authorized/licensed clinician. Medications to be reported in the Case Report Form (CRF) are concomitant prescription medications, over-the-counter medications and supplements. Concomitant therapy will be monitored at all study visits as outlined in the Schedule of Activities, **Section 1.3**. Concomitant medications prohibited during this study include growth hormone, aromatase inhibitors, gonadotropin releasing hormone analogs, recombinant IGF-1, oxandrolone, testosterone, and all forms of estrogen treatment.

6.5.1 RESCUE MEDICINE

Not applicable

7 STUDY INTERVENTION DISCONTINUATION AND PARTICIPANT DISCONTINUATION/WITHDRAWAL

7.1 DISCONTINUATION OF STUDY INTERVENTION

Discontinuation from vosoritide does not mean discontinuation from the study, and remaining study procedures should be completed as indicated by the study protocol. If a clinically significant finding is identified (including, but not limited to changes from baseline) after enrollment, the investigator or qualified designee will determine if any change in participant management is needed. Any new clinically relevant finding will be reported as an adverse event (AE).

The data to be collected at the time of study intervention discontinuation are outlined in the Early Termination visit in the Schedule of Activities, **Section 1.3**.

Individual Subject Stopping Criteria

Dosing for the subject will be temporarily discontinued and DSMB will be informed (at a minimum) if any of the following occur:

• Any treatment emergent adverse event (TEAE) at least Grade 3 (according to the most current version of the CTCAE) assessed by the PI determined to be related to the study drug
• Any two TEAEs Grade 2 experienced by the same subject within one week (according to the CTCAE) including 2 symptomatic hypotension events within one week (non-urgent medical intervention indicated) or any Grade 3 hypotension event (medical intervention or hospitalization indicated) assessed by the PI. Two grade 2 injection site reactions such as presence of swelling at the injection site will not require urgent reporting to the DSMB or subject suspension as long as no further intervention was required.
• Prolongation of QTcF (QT corrected Fredericia) interval to more than 500 msec, ventricular tachycardia greater than five beats
• Any clinically significant worsening of existing disproportionate growth, as determined by physical measurement ratios and/or PI assessment based on measurement or clinical observation findings
o Sitting height to standing height ratio – Worsening of ratio of more than 1 standard deviation from initiation of therapy based on NHANES data [49].
o Arm span minus height – Worsening of more than 1 standard deviation since initiation of therapy with a SDS either >+2 or <-2. Data is based on Gerver et al[50].
• Excessively rapid increase in growth velocity (defined as greater than the 90th percentile on age- and gender-specific growth charts) sustained over a period of 2 years or associated with an adverse event such as joint pains or deformity [51]
• Clinically significant worsening of scoliosis as observed via clinical or radiographic assessment. This is defined as a new onset asymmetry noticed during a forward bend test or an increase in Cobb angle of >10 degrees from baseline or any Cobb angle of >20 degrees at any point in the study.

Subjects may continue in the extension phase of the study until achieving near final adult height which will be defined as either:

- A growth velocity <1.5 cm/year over the prior 12 months
- A bone age via the Greulich and Pyle method of 13 years in females and 15 years in males.

After PI consultation with the DSMB, the subject may be re-challenged with a 20% dose reduction for any AE which may be dose related and is reversible with dose decrease (such as hypotension). If excessive increase in growth, worsening of scoliosis or other conditions related to bone overgrowth are noted, vosoritide will be discontinued permanently, and the subject will be followed-up off treatment for the duration of the study. If the subject meets stopping criteria on re-challenge or if the subject refuses re-challenge, the subject will be permanently discontinued from the medication with ongoing assessment in the study.

Study Suspension Criteria

Dosing for all subjects will be temporarily discontinued and DSMB will be informed (at a minimum) if any of the following occur:

• Any two subjects with a TEAE at least Grade 3 (according to the most current version of the CTCAE) assessed by the PI to be related to study drug

• Any two subjects with two TEAEs Grade 2 experienced by the same subject within one week (according to the CTCAE) including 2 symptomatic hypotension events within one week (non-urgent medical intervention indicated) or any Grade 3 hypotension event (medical intervention or hospitalization indicated) assessed by the PI. Two grade 2 injection site reactions such as presence of swelling at the injection site will not meet this criterion as long as no further intervention was required.
• Any two subjects with prolongation of QTc interval to more than 500 msec, ventricular tachycardia greater than five beats
• Any two subjects with clinically significant worsening of existing disproportionate growth as defined above in the individual stopping criteria
• Any two subjects with excessively rapid increase in growth velocity (defined as greater than the 90th percentile on age- and gender-specific growth charts) sustained over a period of 2 years or associated with an adverse event such as joint pains or deformity which required discontinuation of study drug [51]
• Any two subjects with clinically significant worsening of scoliosis as as defined above in the individual stopping criteria
• Any other reason the DSMB advises temporary discontinuation of cohort dosing until further review of safety data is conducted

The DSMB will review available, relevant safety data within 5-7 days after the PI is apprised of an event. Based on its review, the DSMB may make any of the following recommendations:
• Continue dosing of subjects as planned with additional safety monitoring and/or safety reviews as indicated
• Decrease dose for all subjects
• Extend temporary treatment discontinuation until additional data are available and/or further review/consultation occurs
• Permanently discontinue treatment for all subjects

7.2 PARTICIPANT DISCONTINUATION/WITHDRAWAL FROM THE STUDY

Subjects (or their legally authorized representative) have the right to withdraw their consent to participate in the study at any time without prejudice. The Investigator must withdraw from the study any subject who requests to be withdrawn. A subject's participation in the study may be discontinued at any time at the discretion of the Investigator and in accordance with his/her clinical judgment. When possible, the tests and evaluations listed for the termination visit should be carried out.

Reasons for which a subject may be withdrawn from the study or from study treatment by the Investigator include but are not limited to the following:
• Subject experiences a serious or intolerable AE
• Subject develops a clinically significant laboratory abnormality
• Subject requires medication or medical procedure prohibited by the protocol
• Subject does not adhere to study requirements specified in the protocol
• Subject was erroneously admitted into the study or does not meet entry criteria
• Subject is lost to follow-up

Subjects may continue in the extension phase of the study until achieving near final adult height which will be defined as either:

- A growth velocity <1.5 cm/year over the prior 12 months

- A bone age via the Greulich and Pyle method of 13 years in females and 15 years in males.

Non-adherence to study medication administration will be monitored via review of the medication administration log and counting of empty vials at the 6 and 12 month visits. Subjects will be encouraged to have 100% compliance with study drug administration. If during a 30 day period, a subject misses 5 or more shots, they will be considered non-compliant with the study protocol. They will be given a warning that compliance must improve or they will be withdrawn from the study. If there is a second 30 day period when the subject misses 5 or more shots, they will be withdrawn from the study.

The reason for participant discontinuation or withdrawal from the study will be recorded on the Termination Visit Case Report Form. Subjects who sign the informed consent form but do not receive vosoritide may be replaced. Subjects who sign the informed consent form, and receive vosoritide, and subsequently withdraw, or are withdrawn or discontinued from the study will not be replaced.

7.3 LOST TO FOLLOW-UP

A participant will be considered lost to follow-up if he or she fails to return for any in-person scheduled visits and is unable to be contacted by the study site staff.

The following actions must be taken if a participant fails to return to the clinic for a required study visit:

- The site will attempt to contact the participant and reschedule the missed visit as soon as possible and counsel the participant on the importance of maintaining the assigned visit schedule and ascertain if the participant wishes to and/or should continue in the study.
- Before a participant is deemed lost to follow-up, the investigator or designee will make every effort to regain contact with the participant (where possible, 3 telephone calls and, if necessary, a certified letter to the participant's last known mailing address or local equivalent methods). These contact attempts will be documented in the participant's study file.
- Should the participant continue to be unreachable, he or she will be considered to have withdrawn from the study with a primary reason of lost to follow-up.

8 STUDY ASSESSMENTS AND PROCEDURES

8.1 EFFICACY ASSESSMENTS

Demographic Data and Medical History

Demographic data and a detailed medical history will be obtained at Screening and again at the Day 1 visit. This medical history should elicit all major illnesses, diagnoses, and surgeries that the subject has ever had, and any prior or existing medical conditions that might interfere with study participation or safety.

Measures of Medication Compliance

The subject's caregiver will demonstrate proper medication preparation and administration technique at the Day 2 Visit. Any deficiencies will be corrected with additional education. Subjects will fill out a daily medication administration log. These will be reviewed during all telephone calls and in person at the 6 and 12 month study visits and all in-person extension phase visits. The log will be compared to the empty vial counts at the 6 and 12 month visits and all in-person extension phase visits.

Parental Anthropometrics

The standing height of the participant's biological parents will be assessed if they agree to participate. Both parents should be measured if available. If unavailable, report of the parents' heights will be recorded.

Subject Anthropometrics

Anthropometric measurements include standing height, weight, sitting height, head circumference and arm span. Standing height should be measured 5 times using a calibrated stadiometer. The average of the 5 measurements will be used as the outcome measure. Sitting height should be measured three times using a seated stadiometer. The average of the 3 measurements will be used as the outcome measure. Weight should be measured on a digital scale. Head circumference and arm span will each be measured once using standard procedures. Sitting height ratio will be calculated as the sitting height divided by the standing height. Arm span minus standing height will also be calculated. All measurements will be performed in centimeters.

Height Outcome Measures

Efficacy will be assessed as the change in annualized growth velocity from the baseline period (screening to Visit Day 1) to the intervention period (Visit Day 1 to 12 Month Visit). Efficacy will also be assessed as the change in height SDS from Visit Day 1 to 12 Month Visit. Height SDS will be calculated using the CDC growth curves and growth velocity SDS will be based on the Bone Mineral Density in Childhood Study [51]. In subjects with hypochondroplasia or *ACAN* mutations, an adequate response to therapy will be considered an increase in annualized growth velocity of $\geq$1.6 cm/year based on the adjusted mean response to vosoritide in the Phase III trial in patients with achondroplasia which is clinically significant[48]. In subjects with a Rasopathy, SHOX mutation, NPR2 mutation or CNP deficiency, an adequate response to therapy will be considered an increase in annualized growth velocity of $\geq$3 cm/year (see details in inclusion criteria). Growth velocity and change in height SDS will be calculated on an annual basis during the extension phase of the study.

Pharmacokinetic Variables

During the Day 1 and 6 and 12 month visits, PK plasma samples are collected pre-dose and at 5 (± 2 min), 15 (± 2 min), 30 (± 5 min), 60 (± 5 min), 90 (± 5 min), 120 (± 5 min) min, and 180 (± 5 min) min post-dose.

The following PK parameters will be estimated by non-compartmental analysis for Day 1 and 6 and 12 Months:

- Area under the plasma concentration-time curve from time 0 to infinity (AUC0-∞)
- Area under the plasma concentration-time curve from 0 to the time of last measurable concentration (AUC0-t)
- Cmax
- Tmax
- Elimination half-life (t1/2)
- Apparent clearance of drug (CL/F)
- Apparent volume of distribution based upon the terminal phase (Vz/F)

Samples for PK markers will be sent to BioMarin or their designated contracted laboratory for analysis. Details of processing and shipping will be in the Laboratory Manual. In brief, the samples will be labeled with a coded ID number as well as with the date and time of collection. The will be placed on ice and serum will be separated. A protease inhibitor will be added to prevent degradation of vosoritide.

Samples will then be stored in Dr. Dauber's research laboratory until shipment. BioMarin or the designated laboratory will return all unused samples to Children's National after completion of PK analyses.

Pharmacodynamic Markers
Samples will be obtained for urinary cGMP, if available, at baseline at the Screening Visit and at baseline and 1 hour, 2 hours and 4 hours post vosoritide injection at the Day 1 Visit, 6 Month Visit and 12 Month Visit. Each time point will have a +- 15 minute window. Children will not be catheterized. If they are unable to void at the specified time, that sample will be skipped. Urine creatinine samples will be obtained at all of the same time points. Serum collagen X marker measurement will be obtained at baseline at the Screening Visit, Day 1 Visit, 6 Month Visit and 12 Month Visit and all in-person extension phase visits. Assays for PD markers will be done at outside research laboratories. Details of the specific laboratories, processing needs, and shipping instructions will be in the Laboratory Manual. Coded samples will be provided with the date and time of collection. Remaining samples will be destroyed after assay completion.

DXA scan
DXA scans will be performed as per routine clinical protocols at Children's National Hospital and will include whole body less head as well as a lumbar spine DXA. The amount of radiation from a standard DXA is 0.013 mSv. DXA scans will be done every 24 months during the extension phase of the trial.

QoLISSY Scale Measurements
QoLISSY, the Quality of Life in Short Stature Youth, is a validated questionnaire that includes modules for both parents and children. We will administer both modules (as age appropriate) at the Screening Visit, Day 1 Visit, 6 Month Visit and 12 Month Visit and all in-person extension phase visits. The instrument will be scored as per the user manual's instructions [52].

8.2 SAFETY AND OTHER ASSESSMENTS

Health events
Health events (medical issues that result in a hospital admission or trip to the emergency department, other significant medical issues, and changes in the participant's health status including changes to prescription medications) will be reviewed at the time points indicated in the Schedule of Activities (**Section 1.3**) and recorded on a case report form (CRF). To assist participants' parents in recalling health events, they will be provided with a Health Events Diary on which they can record health events experienced by their child. Updates to concomitant medications will be captured on the appropriate CRF. Should the occurrence of an adverse event (AE) or serious adverse event (SAE) come to the attention of study personnel during review of heath events, it will be captured on the appropriate CRF.

Physical Examination and Tanner Staging
Physical examination will include assessment of general appearance; cardiovascular; dermatologic; head, eyes, ears, nose, and throat; lymphatic; respiratory; GI; musculoskeletal; and neurological/psychological and genitourinary. Tanner stage will also be assessed. All physical examinations will include a detailed musculoskeletal examination that will include palpation of bone structures and joints for tenderness and assessment of mobility and gait to monitor risk of bone overgrowth. If concerns of bone overgrowth arise, referral will be made to an orthopedist for further clinical evaluation including radiographic evaluation. The Screening Visit results will be the baseline

values and clinically significant changes from baseline will be recorded as an AE or SAE as appropriate. A qualified medical provider (either physician or NP) will perform the physical examination.

Vital Signs
All treatment visits have pre-dose vital sign assessments. Vital signs include: body temperature in degrees Celsius (°C), heart rate, blood pressure, and respiratory rate. On visit Day 1 only, minimum assessment frequency is every 15 min (± 5 min) for 2 hours post dose; and assessment is taken every 30 min (±5 min) from 2 to 4 hours post dose. From 4 to 8 hours post-dose, measurements are taken every 60 min (± 10 min). At the Day 2 Visit, vital signs will be assess every 30 minutes (± 5 min) for 2 hours post dose. Vital sign measurements are taken in a sitting position after at least 5 minutes of rest. Heart rate should be taken at each time point that blood pressure is measured. When blood samples and blood pressure assessments are scheduled at the same time or within the same time window, blood pressure should be measured before blood samples are drawn. Vital signs may be monitored more frequently or for longer duration post-dose as clinically indicated.

ECGs
A standard 12-lead ECG will include heart rate, rhythm, intervals, axis, conduction defects, and anatomic abnormalities. If clinically significant abnormalities are noted, the Investigator or designee is required to assess whether it is appropriate for the subject to continue in the study. ECGs will be performed at the time points indicated in the Schedule of Activities (**Section 1.3**). All ECGs will be reviewed by a study investigator and if any questions arise, they will be reviewed by a cardiologist at Children's National Hospital.

Concomitant Medication Assessment
A list of all medications will be assessed at the time points indicated in the Schedule of Activities (**Section 1.3**). The names of the medications will be recorded in a case report form. Dosing information will not be collected.

Echocardiogram
Cardiac anatomy and function will be evaluated by a standard 2-dimensional Doppler ECHO.
The data recorded is to include ventricular cavity size, valve characterization (presence or absence of valve stenosis or regurgitation and clinical significance), ventricular wall thickness, regional wall motion, LV mass calculation, and pericardial characterization. Echocardiograms will be performed at the time points indicated in the Schedule of Activities (**Section 1.3**).

Clinical Labs and Tissue Transglutaminase Antibody
Clinical labs include: complete blood count with differential, complete metabolic panel, lipid panel, TSH, free T4, IGF-I, IGFBP-3, prolactin. All labs will be obtained through the clinical lab at Children's National Hospital which is a CLIA approved lab. Additionally, an extra 10 cc of blood will be obtained for storage for future analysis. This extra blood will be spun down for serum and plasma. The tubes will have the ID code as well as the date and time of collection recorded. These samples will be stored in Dr. Dauber's research laboratory at Children's National. Clinical labs will be performed at the time points indicated in the Schedule of Activities (**Section 1.3**). A Tissue Transglutaminase IgA antibody will be sent to the Children's National Hospital clinical lab at the Screening Visit as an assessment for celiac disease. Details of all laboratory specimen collections will be provided in the Laboratory Manual.

Anti-Vosoritide Immunogenicity Assessments

Blood serum sampling for immunogenicity assessment will be performed at the time points indicated in the Schedule of Activities (**Section 1.3**). Total anti-BMN 111 immunogenicity analysis (TAb) and neutralizing antibodies (NAb) will be collected from serum. Samples will be collected prior to administration of vosoritide. TAb will be measured on all samples as indicated in the table. NAb will be stored and testing of NAb will only be triggered when a decline in cGMP signal is observed in the cGMP biomarker assay at both the 6 and 12 month visits in a TAb positive subject. Subjects with positive neutralizing antibodies will be monitored until the antibody levels return to baseline. Immunogenicity testing will be coordinated through BioMarin. Details of sample processing and shipping will be provided in the Lab Manual.

Bone Age and Spine X-rays

Bone age and Spine X-rays will be performed using routine clinical protocols. The bone age X-rays will be done at an interval similar to routine clinical care. The spine X-ray will be a posteroanterior view of the spine. If the clinical examination suggests a new onset spine asymmetry, an additional spine X-ray will be performed at the 6 month visit. The radiation dose from a single spine X-ray is 1.5 mSv. For the inclusion criterion, the official clinical read of the bone age X-ray will be used to determine eligibility. At the conclusion of the study, all bone age X-rays will be read by a single centralized reader who is blinded to the age of the child and the time point that the X-ray was taken. The single reader will be either a radiologist or endocrinologist at Children's National. The images will only be labeled with the study ID. The ratio of bone age/chronological age will be calculated based on these values.

Adverse Events

The occurrence of AEs will be assessed continuously from the time the subject signs the ICF. The determination, evaluation and reporting of AEs will be performed as outlined in Section 8.3. Assessments of AEs will occur at the time points shown in the Schedule of Activities (Section 1.3).

NOTE: These safety assessments (except for X-rays) may be done at additional time points at the discretion of the PI in order to monitor for the development of adverse events or to ensure that the subject's baseline status has not changed which would preclude participation. X-rays are excluded as they increase the radiation exposure. For example, extra echocardiograms may be ordered if recommended by the study cardiologist.

Safety Imaging During the Extension Phase

The extension phase of the study may last many years as subjects enrolled at age 3 years could have up to 15 years until growth cessation. We will conduct yearly bone age and DXA assessments every 2 years as part of the follow up evaluation. However, we will not do additional routine imaging in order to avoid excessive radiation exposure. If there is concern for a musculoskeletal issue (e.g. worsening scoliosis, bone pain or deformity), additional imaging will be ordered by the investigator as clinically appropriate to assess the concern. Additionally, routine follow up echocardiograms will not be performed after the 12 Month Visit. However, if the study cardiologist has any concerns based on the echocardiogram done at the 12 Month Visit, she may recommend additional echocardiograms at her discretion.

8.3 ADVERSE EVENTS AND SERIOUS ADVERSE EVENTS

8.3.1 DEFINITION OF ADVERSE EVENTS (AE)

Adverse event means any untoward medical occurrence associated with the use of an intervention in humans, whether or not considered intervention-related (21 CFR 312.32 (a)).

For this protocol, a reportable AE is any untoward medical occurrence (eg, sign, symptom, illness, disease or injury) in a subject administered the study-drug or other protocol-imposed intervention, regardless of attribution. This includes the following:

- AEs not previously observed in the subject that emerge during the course of the study.
- Pre-existing medical conditions judged by the Investigator to have worsened in severity or frequency or changed in character during the study.
- Complications that occur as a result of non-drug protocol-imposed interventions (e.g., AEs related to screening procedures, medication washout, or no-treatment run-in).
- Clinically significant post-screening test results that require medical intervention.

An adverse drug reaction is any AE for which there is a reasonable possibility that the study drug caused the AE. “Reasonable possibility” means there is evidence to suggest a causal relationship between the study-drug and the AE.

After informed consent but prior to initiation of vosoritide, only SAEs associated with any protocol-imposed interventions will be reported.

8.3.2 DEFINITION OF SERIOUS ADVERSE EVENTS (SAE)

An adverse event (AE) or suspected adverse reaction is considered "serious" if, in the view of the investigator/sponsor, it results in any of the following outcomes: death, a life-threatening adverse event, inpatient hospitalization or prolongation of existing hospitalization, a persistent or significant incapacity or substantial disruption of the ability to conduct normal life functions, or a congenital anomaly/birth defect. Important medical events that may not result in death, be life-threatening, or require hospitalization may be considered serious when, based upon appropriate medical judgment, they may jeopardize the participant and may require medical or surgical intervention to prevent one of the outcomes listed in this definition. Examples of such medical events include allergic bronchospasm requiring intensive treatment in an emergency room or at home, blood dyscrasias or convulsions that do not result in inpatient hospitalization, or the development of drug dependency or drug abuse.

8.3.3 CLASSIFICATION OF AN ADVERSE EVENT

8.3.3.1 SEVERITY OF EVENT

The Investigator will determine the severity of each AE using the most current version of the Common Terminology Criteria for Adverse Events (CTCAE). Adverse events that do not have a corresponding CTCAE term will be assessed according to the following guidelines:

- **Mild** – Events require minimal or no treatment and do not interfere with the participant’s daily activities.
- **Moderate** – Events result in a low level of inconvenience or concern with the therapeutic measures. Moderate events may cause some interference with functioning.
- **Severe** – Events interrupt a participant’s usual daily activity and may require systemic drug therapy or other treatment. Severe events are usually potentially life-threatening or incapacitating. Of note, the term “severe” does not necessarily equate to “serious”.

8.3.3.2 RELATIONSHIP TO STUDY INTERVENTION

All adverse events (AEs) must have their relationship to study intervention assessed by the investigator based on temporal relationship and his/her clinical judgment. The degree of certainty about causality will be graded using the categories below. In a clinical trial, the study product must always be suspect.

- **Definitely Related** – There is clear evidence to suggest a causal relationship, and other possible contributing factors can be ruled out. The clinical event, including an abnormal laboratory test result, occurs in a plausible time relationship to study intervention administration and cannot be explained by concurrent disease or other drugs or chemicals. The response to withdrawal of the study intervention (dechallenge) should be clinically plausible. The event must be pharmacologically or phenomenologically definitive, with use of a satisfactory rechallenge procedure if necessary.
- **Probably Related** – There is evidence to suggest a causal relationship, and the influence of other factors is unlikely. The clinical event, including an abnormal laboratory test result, occurs within a reasonable time after administration of the study intervention, is unlikely to be attributed to concurrent disease or other drugs or chemicals, and follows a clinically reasonable response on withdrawal (dechallenge). Rechallenge information is not required to fulfill this definition.
- **Potentially Related** – There is some evidence to suggest a causal relationship (e.g., the event occurred within a reasonable time after administration of the trial medication). However, other factors may have contributed to the event (e.g., the participant's clinical condition, other concomitant events). Although an AE may rate only as "possibly related" soon after discovery, it can be flagged as requiring more information and later be upgraded to "probably related" or "definitely related", as appropriate.
- **Unlikely to be related** – A clinical event, including an abnormal laboratory test result, whose temporal relationship to study intervention administration makes a causal relationship improbable (e.g., the event did not occur within a reasonable time after administration of the study intervention) and in which other drugs or chemicals or underlying disease provides plausible explanations (e.g., the participant's clinical condition, other concomitant treatments).
- **Not Related** – The AE is completely independent of study intervention administration, and/or evidence exists that the event is definitely related to another etiology. There must be an alternative, definitive etiology documented by the clinician.

8.3.3.3 EXPECTEDNESS

The PI will be responsible for determining whether an adverse event (AE) is expected or unexpected. An AE will be considered unexpected if the nature, severity, or frequency of the event is not consistent with the risk information previously described for the study intervention.

8.3.4 TIME PERIOD AND FREQUENCY FOR EVENT ASSESSMENT AND FOLLOW-UP

The occurrence of an adverse event (AE) or serious adverse event (SAE) may come to the attention of study personnel during study visits or phone contacts with a study participant. Assessments for AE will occur as outlined in the Schedule of Activities (**Section 1.3**).

All AEs including local and systemic reactions not meeting the criteria for SAEs will be captured on the appropriate case report form (CRF). Information to be collected includes event description, time of onset, clinician's assessment of severity, relationship to study product (assessed only by those with the training and authority to make a diagnosis), and time of resolution/stabilization of the event. All AEs occurring while on study must be documented appropriately regardless of relationship. All AEs will be

followed until the event has resolved or any permanent sequelae are stable. The study team will work with the local clinicians to determine the characterization and severity of an AE and the proper course of treatment, if appropriate.

Any medical condition that is present at the time that the participant is screened will be considered as baseline and not reported as an AE. However, if the study participant's condition deteriorates at any time during the study, it will be recorded as an AE.

Changes in the severity of an AE will be documented to allow an assessment of the duration of the event at each level of severity to be performed. AEs characterized as intermittent require documentation of onset and duration of each episode.

The study coordinator, with supervision of the PI, will record all reportable events with start dates occurring any time after informed consent is obtained until 7 (for non-serious AEs) or 30 days (for SAEs) after the last day of study participation. At each study visit, the investigator will inquire about the occurrence of AE/SAEs since the last visit. Events will be followed for outcome information until resolution or stabilization. For events that are not resolved or stable at the last study visit, the study team will continue to contact the family by phone on a weekly basis until resolution or stabilization.

8.3.5 ADVERSE EVENT REPORTING

Subjects will be encouraged to notify the study team immediately should any adverse event occur. Formal review of adverse events will occur at each visit and telephone call as detailed in the Schedule of Activities (**Section 1.3**). The PI or a qualified co-investigator will review all adverse events within 48 hours and serious adverse events within 24 hours of becoming aware of the event or as soon as possible if clinical treatment or dose reduction may be necessary. An adverse event log will be maintained. Reports of all AEs will be provided to the DSMB after completion of Visit Day 1 for the first 5 subjects and then after completion of the Day 1 Visits for each additional 10 subjects (i.e. after subjects 15, 25, and 35, etc…) or every 6 months, whichever is sooner. The study team will comply with all local legal, regulatory, and IRB requirements with regards to adverse events.

8.3.6 SERIOUS ADVERSE EVENT REPORTING

All study personnel will immediately report to the sponsor investigator any serious adverse event (SAE), whether or not considered study intervention related, including those listed in the protocol or investigator brochure and must include an assessment of whether there is a reasonable possibility that the study intervention caused the event. The PI will report all SAEs to BioMarin within 24 hours of being made aware of an occurrence using FDA form 3011A.

All SAEs will be followed until satisfactory resolution or until the PI deems the event to be chronic or the participant is stable.

The study sponsor/PI will be responsible for notifying the Food and Drug Administration (FDA) of any unexpected fatal or life-threatening suspected adverse reaction as soon as possible, but in no case later than 7 calendar days after the sponsor's initial receipt of the information. In addition, the sponsor investigator must notify FDA and BioMarin must notify all participating investigators in an Investigational New Drug (IND) safety report of potential serious risks, from clinical trials or any other source, as soon as possible, but in no case later than 15 calendar days after the sponsor investigator determines that the information qualifies for reporting. The IND safety report will be submitted to the FDA and to BioMarin

using Form 3500A as per FDA regulations and will follow the appropriate FDA guidance (see https://www.fda.gov/drugs/investigational-new-drug-ind-application/ind-application-reporting-safety-reports).

All reports to BioMarin will be address to BioMarin's Drug Safety Department is as follows:

BioMarin Pharmaceutical Inc.
105 Digital Drive
Novato, CA 94949
Phone: (415) 506-6179
Fax: (415) 532-3144
Email: drugsafety@bmrn.com

8.3.7 REPORTING EVENTS TO PARTICIPANTS

AEs or SAEs will be reported to all participants via email and mail if either the IRB determines that this information changes the risk benefit considerations for the trial or if reporting is mandated by the DSMB. An amendment will be submitted to the IRB containing the relevant new information and any applicable informed consent updates.

8.3.8 EVENTS OF SPECIAL INTEREST

Not applicable

8.3.9 REPORTING OF PREGNANCY

Not applicable

8.4 UNANTICIPATED PROBLEMS

8.4.1 DEFINITION OF UNANTICIPATED PROBLEMS (UP)

The Office for Human Research Protections (OHRP) considers unanticipated problems involving risks to participants or others to include, in general, any incident, experience, or outcome that meets **all** of the following criteria:

- Unexpected in terms of nature, severity, or frequency given (a) the research procedures that are described in the protocol-related documents, such as the Institutional Review Board (IRB)-approved research protocol and informed consent document; and (b) the characteristics of the participant population being studied;
- Related or possibly related to participation in the research ("possibly related" means there is a reasonable possibility that the incident, experience, or outcome may have been caused by the procedures involved in the research); and
- Suggests that the research places participants or others at a greater risk of harm (including physical, psychological, economic, or social harm) than was previously known or recognized.

8.4.2 UNANTICIPATED PROBLEM REPORTING

The investigator will report unanticipated problems (UPs) to the Institutional Review Board (IRB) and to the DSMB. The UP report will include the following information:

- Protocol identifying information: protocol title and number, PI's name, and the IRB project number;
- A detailed description of the event, incident, experience, or outcome;
- An explanation of the basis for determining that the event, incident, experience, or outcome represents an UP;
- A description of any changes to the protocol or other corrective actions that have been taken or are proposed in response to the UP.

To satisfy the requirement for prompt reporting, UPs will be reported using the following timeline:

> UPs will be promptly report to the Children's National IRB and DSMB within 7 business days of the investigator becoming aware of the UP. In the case of a participant death, the IRB and DSMB will be notified within 24 hours.

8.4.3 REPORTING UNANTICIPATED PROBLEMS TO PARTICIPANTS

UPs will be reported to all participants via email and mail if either the IRB determines that this information changes the risk benefit considerations for the trial or if reporting is mandated by the DSMB.

9 STATISTICAL CONSIDERATIONS

The statistical analysis plan (SAP) will provide additional details on the planned statistical analysis.

9.1 STATISTICAL HYPOTHESES

- Primary Safety Endpoint(s): We will rule out safety concerns. That is, we will rule out the null hypothesis that risk of adverse events post-drug administration will be more than twice the risk during the baseline period (i,e, RR or OR > 2).

- Primary Efficacy Endpoint(s): The null hypothesis is that there is no change from baseline in annualized growth velocity or height SDS over the 12 months of intervention when compared to the 6 month baseline observation period. The alternate hypothesis is that there is a change from baseline in these values.

- Secondary Efficacy Endpoint(s): The null hypothesis is that there is no change from baseline in seated height ratio, arm span minus standing height, or bone age divided by chronological age over the 12 months of intervention when compared to the 6 month baseline observation period. The alternate hypothesis is that there is a change from baseline in these values.

9.2 SAMPLE SIZE DETERMINATION

This is an exploratory proof of principle Phase I/II study, and therefore, no formal sample size calculations were performed. The study size was chosen based on the feasibility of recruitment in this study time period.
Once the initial sample size of 35 subjects is achieved, we will discontinue recruitment of any genetic cohort with greater than 15 subjects. Additional recruitment efforts will then focus on achieving approximately 10 subjects in each of the cohorts.

9.3 POPULATIONS FOR ANALYSES

The analysis population for all efficacy and safety analysis is all subjects who receive at least one dose of study treatment.

9.4 STATISTICAL ANALYSES

9.4.1 GENERAL APPROACH

Analyses on all endpoints will be self-controlled, comparing measurements for each subject during follow-up to the same measurements during the baseline period. Thus, all measurements will be summarized for baseline period (control period), and during follow-up (test drug period). Descriptive statistics will be presented for all measures of change from baseline (mean, standard deviation, median, interquartile range, min and max). Comparisons between baseline period and follow-up period will use a paired t-test for continuous endpoints and a chi-square test for dichotomous endpoints. If there are departures from normality or small samples, we will use Mann-Whitney test for comparison of medians and exact Clopper-Pearson test for comparison of dichotomous endpoints.

9.4.2 ANALYSIS OF THE PRIMARY EFFICACY ENDPOINT(S)

Growth velocity, based on anthropometric measures, at each scheduled time point will be summarized using descriptive statistics (mean, SD, median, interquartile range, minimum, and maximum). The test of hypothesis of no change from the baseline in growth velocity and height SDS will be conducted via paired t-test. The measurement of height will be converted to age-and sex-appropriate standard score (SDS) by comparison with the CDC reference standards. The Z-score will be summarized similarly to growth velocity. Additional details of the analysis including management of missing data, and approach to handling outliers, non-adherent and lost to follow up subject data will all be included in a separate statistical analysis plan.

9.4.3 ANALYSIS OF THE SECONDARY ENDPOINT(S)

Change in sitting height ratio, arm span minus standing height, and bone age/chronological age ratio from baseline to each scheduled time point will be summarized and tested similarly to the primary efficacy analyses. Additional details of the analysis including management of missing data, and approach to handling outliers, non-adherent and lost to follow up subject data will all be included in a separate statistical analysis plan.

9.4.4 SAFETY ANALYSES

All subjects who receive at least one dose of study treatment in this study will be included in the end-of-study safety analysis. The end-of-study safety analysis will be descriptive.

All AEs will be categorized and classified for severity as described above based on the original terms entered on the CRF. The incidence of AEs will be summarized by system organ class, preferred term, relationship to study treatment, and severity. All AEs, including AEs that lead to dose interruption/hold, permanent discontinuation from the study and from study treatment and SAEs, will be listed.

All other safety measures including laboratory tests, vital signs, ECG, and concomitant medication data will also be summarized descriptively. Laboratory tests will also be summarized by absolute and percent change from baseline and listed by significant values.

9.4.5 BASELINE DESCRIPTIVE STATISTICS

Descriptive statistics of the cohort will be based on the 6 month observation period. These will include baseline height velocity, height SDS, body proportions, quality of life score, and adverse events. They will be described as noted in Section 9.4.1 above.

9.4.6 PLANNED INTERIM ANALYSES

There are no planned interim analyses. The study will only be discontinued if safety stopping criteria described in **Section 7.1** are met.

9.4.7 SUB-GROUP ANALYSES

Analyses will be stratified by the following subgroups: age, sex, and genetics (presence of a pathogenic or likely pathogenic variant in an individual genetic subgroup).

9.4.8 TABULATION OF INDIVIDUAL PARTICIPANT DATA

Individual participant date will be listed for each measure and time point.

9.4.9 EXPLORATORY ANALYSES

PK parameters will be summarized using descriptive statistics. Changes in bone density Z-score will be analyzed similar to the primary efficacy analyses. Change in cGMP, serum collagen X markers, and changes in QoLISSY will be compared via t-test at each time point compared to baseline. Rates of total anti-vosoritide antibodies and neutralizing antibodies will be reported.

10 SUPPORTING DOCUMENTATION AND OPERATIONAL CONSIDERATIONS

10.1 REGULATORY, ETHICAL, AND STUDY OVERSIGHT CONSIDERATIONS

10.1.1 INFORMED CONSENT PROCESS

10.1.1.1 CONSENT/ASSENT AND OTHER INFORMATIONAL DOCUMENTS PROVIDED TO PARTICIPANTS

Consent forms describing in detail the study intervention, study procedures, and risks are given to the participant and written documentation of informed consent is required prior to any study assessments. The following consent materials are submitted with this protocol:

- Consent form for subject to be completed by guardian
- Upon approval of the initial English consent forms, the consent forms will be translated into Spanish using a hospital approved translation service. These will be added to the protocol in an administrative amendment. If subjects are recruited who require consents in a language other than English or Spanish, the consent forms will be translated into the appropriate language and approved by the IRB prior to proceeding with enrollment.
- Consent addendum for the extension phase for the subject to be completed by the guardian
- Assent form for subjects in the extension phase to be completed by the subject at the first in-person visit after their 12th birthday

10.1.1.2 CONSENT PROCEDURES AND DOCUMENTATION

The study team will receive referrals for potential participants from pediatric endocrinologists and geneticists throughout the United States. We will request a HIPPA waiver from the IRB for screening these referrals for the purposes of subject recruitment.

Consent forms will be Institutional Review Board (IRB)-approved. The participant/legally authorized representative (LAR) will be given a copy of the consent forms to read and review the document in advance of the Screening Visit. A telephone call will be scheduled to review the details of the study and consent in advance of the screening visit. This is a purely informational call and will not replace the in person consent. Subjects who remain interested in participating will then be scheduled for a Screening Visit.

At the Screening Visit, the principal investigator or a qualified physician co-investigator will explain the research study to the participant and answer any questions that may arise. This conversation will take place in a private room. Verbal assent will be conducted for children age 7 and older. Written assent will not be obtained as all subjects are under age 12 years. A verbal explanation will be provided in terms suited to the participant's comprehension of the purposes, procedures, and potential risks of the study and of their rights as research participants. Participants/families/LAR will have the opportunity to carefully review the written consent form and ask questions prior to signing. The participants/family/LAR should have the opportunity to discuss the study with their family or surrogates, or think about it prior to agreeing to participate. The participant will sign the informed consent document prior to any procedures being done specifically for the study. Participants/families/LAR must be informed that participation is voluntary and that they may withdraw from the study at any time, without prejudice. A copy of the informed consent document will be given to the participants/families/LAR for their records. The informed consent discussion will be documented in the research record.

For subjects with a good response to therapy at the 12 Month Visit, they will be given the opportunity to continue into the extension phase of the study. If they choose to continue in the extension phase, they will sign a consent addendum. The same procedures will be used as above. Any subjects who have reached age 12 years will be asked to sign a written assent form at the time of enrollment in the extension phase. If they become 12 years old while in the extension phase, they will be asked to sign the written assent form at their next in-person visit. Subjects who do not assent to continue in the study will be withdrawn from the study.

For subjects who do not speak English, we will follow the same procedures but will use a certified interpreter for all interactions. A consent form in the subject's primary language will be used to document consent after verbally reviewing with the interpreter the entire informed consent document.

10.1.2 STUDY DISCONTINUATION AND CLOSURE

This study may be temporarily suspended or prematurely terminated if there is sufficient reasonable cause. Written notification, documenting the reason for study suspension or termination, will be provided by the PI to study participants, BioMarin, and regulatory authorities. If the study is prematurely terminated or suspended, the Principal Investigator (PI) will promptly inform study participants, the Institutional Review Board (IRB), and BioMarin and will provide the reason(s) for the termination or suspension. Study participants will be contacted, as applicable, and be informed of changes to study visit schedule.

Circumstances that may warrant termination or suspension include, but are not limited to:

- Determination of unexpected, significant, or unacceptable risk to participants
- Insufficient compliance to protocol requirements

- Data that are not sufficiently complete and/or evaluable
- Loss of funding and/or support from BioMarin

Study may resume once concerns about safety, protocol compliance, and data quality are addressed, and satisfy the IRB, DSMB, BioMarin and/or Food and Drug Administration (FDA) as necessary.

10.1.3 DATA CONFIDENTIALITY AND PARTICIPANT PRIVACY

Participant confidentiality and privacy is strictly held in trust by the participating investigators, their staff, and the sponsor(s) and their interventions. This confidentiality is extended to cover testing of biological samples and genetic tests in addition to the clinical information relating to participants. Therefore, the study protocol, documentation, data, and all other information generated will be held in strict confidence. No information concerning the study or the data will be released to any unauthorized third party without prior written approval of the sponsor/investigator and IRB.

All research activities will be conducted in as private a setting as possible, such as a clinic exam room.

The study monitor, other authorized representatives of the sponsor, representatives of the Institutional Review Board (IRB), regulatory agencies or BioMarin may inspect all documents and records required to be maintained by the investigator, including but not limited to, medical records (office, clinic, or hospital) and pharmacy records for the participants in this study. Children's National Hospital will permit access to such records.

The study participant's contact information will be securely stored at Children's National Hospital for internal use during the study. At the end of the study, all records will continue to be kept in a secure location for as long a period as dictated by the FDA, reviewing IRB, Institutional policies, or BioMarin requirements.

Study participant research data will be transmitted to and stored at BioMarin. This will not include the participant's contact or identifying information. Rather, individual participants and their research data will be identified by a unique study identification number. The study data entry and study management systems used by clinical site and by BioMarin will be secured and password protected.

Samples and data may be shared with collaborative laboratories/investigators for research purposes. Information pertaining to these procedures is included in the ICF and will be reviewed with participants during the consent process.

10.1.4 FUTURE USE OF STORED HUMAN SPECIMENS AND DATA

Data collected for this study will be analyzed and stored at the Children's National Hospital. After the study is completed, the coded, archived data will be transmitted to and stored at BioMarin in addition to at Children's National Hospital. Permission to transmit data to BioMarin will be included in the informed consent. The code linking the data and samples to individual identifying information will be kept in a secure folder in a limited access network drive at Children's National. Only authorized study staff will have access to this code.

With the participant's approval, coded biological samples will be stored at Children's National Hospital. These samples could be used to research the causes of short stature, its complications and other conditions for which individuals with short stature are at increased risk, and to improve treatment.

During the conduct of the study, an individual participant can choose to withdraw consent to have biological specimens stored for future research. However, withdrawal of consent with regard to biosample storage may not be possible after the study is completed.

When the study is completed, access to study data and/or samples will be provided through Dr. Dauber's laboratory at Children's National Hospital.

10.1.5 KEY ROLES AND STUDY GOVERNANCE

**Principal Investigator and Medical Monitor**
Andrew Dauber, MD MMSc<br>Chief of Endocrinology
Children's National Hospital
111 Michigan Ave NW, Washington, DC 20010
202-476-1241
adauber@childrensnational.org

As this is a small pilot study, Dr. Dauber will serve as the PI and will oversee all aspects of the study directly. We will have an independent external clinical study monitor contracted from PPD Development, LP. Additionally, there will be an independent external DSMB that will review safety data frequently as detailed in the following section.

There will be a study Steering Committee consisting of Dr. Dauber, Kimberly Boucher, RN (Clinical Research Manager for the Endocrine Division), a biostatistician, and an additional qualified co-investigator. The Steering Committee will review all protocol amendments as well as moderate or severe adverse events and will jointly decide on any necessary protocol adjustments.

Children's National Hospital will serve as the IRB of record for this single site trial.

10.1.6 SAFETY OVERSIGHT

Safety oversight will be under the direction of a Data and Safety Monitoring Board (DSMB) composed of individuals with the appropriate expertise, including biostatistics, growth disorders, and clinical trials conduct. For the purpose of this study, we will use the same independent DSMB as was used for the Phase II study of vosoritide in achondroplasia as those DSMB members are very knowledgeable about the specific risk profile of this medication. Members of the DSMB will be independent from the study conduct and free of conflict of interest, or measures should be in place to minimize perceived conflict of interest. Children's National Hospital will independently contract with this DSMB. The DMSB will operate under the rules of an approved charter that will be written and reviewed at the organizational meeting of the DSMB. At this time, each data element that the DSMB needs to assess will be clearly defined. The DSMB will provide its input to Dr. Dauber and the Children's National IRB as needed. Reporting of all AEs will be provided to the DSMB after completion of Visit Day 1 for the first 5 subjects and then after completion of the Day 1 Visits for each additional 10 subjects (i.e. after subjects 15, 25, and 35, etc…) or every 6 months, whichever is sooner. Meetings will occur at a minimum of every 6 months and more frequently at the DSMB's discretion.

10.1.7 CLINICAL MONITORING

Clinical site monitoring is conducted to ensure that the rights and well-being of trial participants are protected, that the reported trial data are accurate, complete, and verifiable, and that the conduct of the trial is in compliance with the currently approved protocol/amendment(s), with International

Conference on Harmonization Good Clinical Practice (ICH GCP), and with applicable regulatory requirement(s).

- Monitoring for this study will be performed by an independent external monitor contracted from PPD Development, LP. We have confirmed that PPD is able to do source data verification from OpenClinica.
- On-site monitoring will occur at a minimum of every 6 months or every 10 subjects enrolled and more frequently if deemed necessary. Random chart audits of 25% of visits will occur and will include 100% data review for those visits. Additional monitoring will be performed of key outcome variables.
- Dr. Dauber will be provided copies of monitoring reports within 10 business days of the visit.
- Details of clinical site monitoring are documented in a Clinical Monitoring Plan (CMP). The CMP describes in detail who will conduct the monitoring, at what frequency monitoring will be done, at what level of detail monitoring will be performed, and the distribution of monitoring reports.

10.1.8 QUALITY ASSURANCE AND QUALITY CONTROL

We will perform internal quality management of study conduct, data and biological specimen collection, documentation and completion. An individualized quality management plan will be developed to describe our quality management plan.

Quality control (QC) procedures will be implemented beginning with the data entry system and data QC checks that will be run on the database will be generated. Any missing data or data anomalies will be resolved.

Following written Standard Operating Procedures (SOPs), the monitors will verify that the clinical trial is conducted and data are generated and biological specimens are collected, documented (recorded), and reported in compliance with the protocol, International Conference on Harmonization Good Clinical Practice (ICH GCP), and applicable regulatory requirements (e.g., Good Laboratory Practices (GLP), Good Manufacturing Practices (GMP)).

Children's National Hospital will provide direct access to all trial related site, source data/documents, and reports for the purpose of monitoring and auditing by the sponsor, and inspection by local and regulatory authorities.

10.1.9 DATA HANDLING AND RECORD KEEPING

10.1.9.1 DATA COLLECTION AND MANAGEMENT RESPONSIBILITIES

Data collection is the responsibility of the clinical trial staff at Children's National Hospital under the supervision of the PI. The investigator is responsible for ensuring the accuracy, completeness, legibility, and timeliness of the data reported. It is expected that data from all study visits will be entered into the electronic research database within 5 business days and will be reviewed by the PI within 2 weeks.

All paper CRFs will be kept as source documents. Details of all CRFs will be provided in the study manual of operations. All source documents should be completed in a neat, legible manner to ensure accurate interpretation of data.

Hardcopies of the study visit worksheets will be provided for use as source document worksheets for recording data for each participant enrolled in the study. Data recorded in the electronic case report

form (eCRF) derived from source documents should be consistent with the data recorded on the source documents.

Clinical data (including adverse events (AEs), concomitant medications, and expected adverse reactions data) and clinical laboratory data will be entered into OpenClinica, a 21 CFR Part 11-compliant data capture system supported by Children's National. The eCRFs' design will be done by the study staff in conjunction with the study biostatistician and a Children's National data manager. The data system includes password protection and internal quality checks, such as automatic range checks, to identify data that appear inconsistent, incomplete, or inaccurate. Clinical data will be entered directly from the source documents.

10.1.9.2 STUDY RECORDS RETENTION

Study documents should be retained for a minimum of 3 years after the termination of the study. Study documents should be retained for a minimum of 2 years after the last approval of a marketing application in an International Conference on Harmonization (ICH) region and until there are no pending or contemplated marketing applications in an ICH region or until at least 2 years have elapsed since the formal discontinuation of clinical development of the study intervention. These documents should be retained for a longer period, however, if required by local regulations.

10.1.10 PROTOCOL DEVIATIONS

A protocol deviation is defined by institutional policy. It is the responsibility of the site investigator to use continuous vigilance to identify and report deviations. All deviations must be addressed in study source documents and reported to Dr. Dauber. As a result of deviations, corrective actions are to be developed by the study staff and implemented promptly. Protocol deviations and corrective actions must be sent to the Children's National IRB per their policies. The site investigator is responsible for knowing and adhering to the reviewing IRB requirements.

10.1.11 PUBLICATION AND DATA SHARING POLICY

This trial is registered at ClinicalTrials.gov (NCT04219007), and results information from this trial will be submitted to ClinicalTrials.gov. In addition, every attempt will be made to publish results in peer-reviewed journals within 1 year of completion of the trial. Data from this study may be requested from other researchers after publication of the initial study results by contacting Dr. Dauber.

10.1.12 CONFLICT OF INTEREST POLICY

The independence of this study from any actual or perceived influence, such as by the pharmaceutical industry, is critical. Therefore, any actual conflict of interest of persons who have a role in the design, conduct, analysis, publication, or any aspect of this trial will be disclosed and managed. Furthermore, persons who have a perceived conflict of interest will be required to have such conflicts managed in a way that is appropriate to their participation in the design and conduct of this trial. The study leadership in conjunction with Children's National Hospital has established policies and procedures for all study group members to disclose all conflicts of interest and will establish a mechanism for the management of all reported dualities of interest.

This is an investigator-initiated academic study which is being supported financially and through provision of study drug by BioMarin. All decisions about study design, study conduct, data analysis, and publication of trial results will be made independently by the study team at Children's National Hospital. BioMarin will be provided with copies of the protocol, data, and planned publications. BioMarin may

comment on these items but all decisions regarding these items will be made by Dr. Dauber and the study team. A clinical trial agreement outlining these terms will be in effect prior to study initiation.

10.2 ADDITIONAL CONSIDERATIONS

Not applicable

10.3 ABBREVIATIONS

AE	Adverse Event
ACAN	Aggrecan
BMD	Bone Mineral Density
CFR	Code of Federal Regulations
CLIA	Clinical Laboratory Improvement Amendments
CNP	C-type Natriuretic Peptide
COC	Certificate of Confidentiality
CONSORT	Consolidated Standards of Reporting Trials
CRF	Case Report Form
CTCAE	Common Terminology Criteria for Adverse Events
DSMB	Data Safety Monitoring Board
FDA	Food and Drug Administration
FGFR3	Fibroblast Growth Factor Receptor 3
GCP	Good Clinical Practice
GLP	Good Laboratory Practices
GMP	Good Manufacturing Practices
GWAS	Genome-Wide Association Studies
HIPAA	Health Insurance Portability and Accountability Act
IB	Investigator’s Brochure
ICH	International Conference on Harmonization
IHH	Indian Hedgehog Signaling Molecule
IND	Investigational New Drug Application
IRB	Institutional Review Board
MOP	Manual of Procedures
MSDS	Material Safety Data Sheet
NAb	Neutralizing Antibodies
NCT	National Clinical Trial
NPPC	Natriuretic Peptide Precursor C
NPR2	Natriuretic Peptide Receptor 2
OHRP	Office for Human Research Protections
PI	Principal Investigator
QA	Quality Assurance
QC	Quality Control
SAE	Serious Adverse Event
SAP	Statistical Analysis Plan
SC	Subcutaneous
SDS	Standard Deviation Score
SHOX	Short Stature Homeobox
SOA	Schedule of Activities
SOP	Standard Operating Procedure
TAb	Total Antibodies
TEAE	Treatment emergent adverse event
UP	Unanticipated Problem

US	United States

10.4 PROTOCOL AMENDMENT HISTORY

The table below is intended to capture changes of IRB-approved versions of the protocol, including a description of the change and rationale. A Summary of Changes table for the current amendment is located in the Protocol Title Page.

Version	Date	Description of Change	Brief Rationale
2.2	July 28, 2020	Addition of monthly phone calls for review of health events during the observation period<br><br>Update to description of extra 10 cc of blood to be collected and stored for future analysis which now reflects that the extra blood will be spun down for both serum (included in previous version) and plasma	Assess safety during baseline period.<br><br>Allow for flexibility in use of stored blood specimens.
2.3	December 23, 2020	Clarified that patients with SHOX gene mutations can have heterozygous or homozygous or compound heterozygous mutations.<br><br>IGF-1 and baseline laboratory evaluations may be repeated during screening period.<br><br>Additional details added to description of drug product and return and accountability procedures.<br><br>Additional and/or repeat safety studies may be added at investigator's discretion.	There was no rationale to include only heterozygous subjects. This was an error in the initial protocol.<br><br>Mild deviations in these screening laboratory studies are expected and if normalize prior to administration of vosoritide should not exclude the subject from participating.<br><br>Updated to include details on Vosoritide drug product configuration.<br><br>Additional safety monitoring may be warranted for mild abnormalities in routine labs or echocardiogram screening.
2.4	February 18, 2021	Addition of subjects with heterozygous mutations in *ACAN* (the gene that encodes Aggrecan) to the inclusion criteria with rationale provided in the background section.	Additional literature review has shown that aggrecan deficiency leads to increased ERK signaling and that CNP therapy can increase production of extracellular matrix in the growth plate. This data supports the use of vosoritide in subjects with aggrecan deficiency.
2.6	November 6, 2021	Addition of extension phase to protocol to allow subjects with a good response to therapy to continue on vosoritide. The protocol was adjusted throughout to reflect the addition of this extension phase. The schedule of activities was updated.<br>Clarification of subject discontinuation criteria based on disproportionality, excessive growth as well as for the end of the extension phase of the study.	Subjects who are responding to the vosoritide therapy will benefit from additional years of therapy until growth cessation. An extension phase will allow the study to assess longer-term safety and efficacy in these subjects.<br>Due to the addition of the extension phase, additional details were needed for subject discontinuation criteria. Additionally, clinical

			experience pointed to the need to refine the disproportionality criteria to highlight that only worsening of disproportionality outside of the normal range and not improvement in disproportionality would lead to subject discontinuation. Additionally, as subjects may demonstrate an initial phase of rapid growth, we clarified that the rapid growth needs to be sustained or associated with an adverse event to lead to dose reduction or discontinuation.
2.7	January 6, 2022	Changed the definition of a positive response to vosoritide to separate it based on genetic etiology.	This change was made after discussion with the FDA as the study includes distinct populations that should have different response criteria.
2.8	September 28, 2022	Adjusted sample size to up to 75 subjects. Clarified that once 35 subjects have been recruited, we will close all cohorts with >15 subjects and focus recruitment efforts to achieve approximately 10 subjects in each genetic cohort<br><br>Clarified that two grade 2 injection site reactions which self-resolve with not require urgent reporting to DSMB or subject suspension from the study as long as no further intervention was required	We seek to provide proof of principle of efficacy in each genetic cohort and thus need a sample of ~10 subjects per cohort to have more robust pilot data.<br><br>According to the current CTCAE version, injection site swelling is a Grade 2 reaction. It was not our intention to require study drug suspension or urgent reporting for injection site swelling which self-resolves.
2.9	May 2, 2023	Updated protocol to state the vosoritide no longer needs to be shipped under cold conditions and temperature monitoring is no longer required during shipment or in the patient's home	The manufacturer has updated drug stability information and vosoritide is now FDA approved without these shipping or monitoring requirements.

11 REFERENCES

1. Bakker, B., Frane, J., Anhalt, H., Lippe, B. & Rosenfeld, R. G. Height Velocity Targets from the National Cooperative Growth Study for First-Year Growth Hormone Responses in Short Children. *J. Clin. Endocrinol. Metab.* **93**, 352–357 (2008).
2. Savarirayan, R. *et al.* C-Type Natriuretic Peptide Analogue Therapy in Children with Achondroplasia. *N. Engl. J. Med.* **381**, 25–35 (2019).
3. Jee, Y. H., Andrade, A. C., Baron, J. & Nilsson, O. Genetics of Short Stature. *Endocrinol. Metab. Clin. North Am.* **46**, 259–281 (2017).
4. Dauber, A., Rosenfeld, R. G. & Hirschhorn, J. N. Genetic Evaluation of Short Stature. *J. Clin. Endocrinol. Metab.* **99**, 3080–3092 (2014).
5. Baron, J. *et al.* Short and tall stature: a new paradigm emerges. *Nat. Rev. Endocrinol.* **11**, 735–746 (2015).
6. Hauer, N. N. *et al.* Clinical relevance of systematic phenotyping and exome sequencing in patients with short stature. *Genet. Med.* **20**, 630–638 (2018).
7. Plachy, L. *et al.* High Prevalence of Growth Plate Gene Variants in Children With Familial Short Stature Treated With GH. *J. Clin. Endocrinol. Metab.* **104**, 4273–4281 (2019).
8. Ornitz, D. M. & Marie, P. J. Fibroblast growth factor signaling in skeletal development and disease. *Genes and Development* vol. 29 1463–1486 (2015).
9. BioMarin. *BMN 111 Investigator's Brochure Version 12.0*. (2020).
10. Hisado-Oliva, A. *et al.* Mutations in C-natriuretic peptide (NPPC): a novel cause of autosomal dominant short stature. *Genet. Med.* **20**, 91–97 (2018).
11. Fujii, T. *et al.* Circulating C-type natriuretic peptide (CNP) rescues chondrodysplastic CNP knockout mice from their impaired skeletal growth and early death. *Endocrinology* **151**, 4381–8 (2010).
12. Bober, M. B., Bellus, G. A., Nikkel, S. M. & Tiller, G. E. *Hypochondroplasia*. *GeneReviews®* (University of Washington, Seattle, 1993).
13. Lorget, F. *et al.* Evaluation of the therapeutic potential of a CNP analog in a Fgfr3 mouse model recapitulating achondroplasia. *Am. J. Hum. Genet.* **91**, 1108–14 (2012).
14. Wendt, D. J. *et al.* Neutral endopeptidase-resistant C-type natriuretic peptide variant represents a new therapeutic approach for treatment of fibroblast growth factor receptor 3-related dwarfism. *J. Pharmacol. Exp. Ther.* **353**, 132–49 (2015).
15. Yasoda, A. *et al.* Systemic administration of C-type natriuretic peptide as a novel therapeutic strategy for skeletal dysplasias. *Endocrinology* **150**, 3138–3144 (2009).
16. Krejci, P. *et al.* Analysis of STAT1 activation by six FGFR3 mutants associated with skeletal dysplasia undermines dominant role of STAT1 in FGFR3 signaling in cartilage. *PLoS One* **3**, (2008).
17. Bartels, C. F. *et al.* Mutations in the transmembrane natriuretic peptide receptor NPR-B impair skeletal growth and cause acromesomelic dysplasia, type Maroteaux. *Am. J. Hum. Genet.* **75**, 27–34 (2004).
18. Olney, R. C. *et al.* Heterozygous mutations in natriuretic peptide receptor-B (NPR2) are associated with short stature. *J. Clin. Endocrinol. Metab.* **91**, 1229–1232 (2006).
19. Vasques, G. A. *et al.* Heterozygous Mutations in Natriuretic Peptide Receptor-B (*NPR2*) Gene as a Cause of Short Stature in Patients Initially Classified as Idiopathic Short Stature. *J. Clin. Endocrinol. Metab.* **98**, E1636–E1644 (2013).
20. Amano, N. *et al.* Identification and Functional Characterization of Two Novel *NPR2* Mutations in Japanese Patients With Short Stature. *J. Clin. Endocrinol. Metab.* **99**, E713–E718 (2014).
21. Hisado-Oliva, A. *et al.* Heterozygous *NPR2* Mutations Cause Disproportionate Short Stature, Similar to Léri-Weill Dyschondrosteosis. *J. Clin. Endocrinol. Metab.* **100**, E1133–E1142 (2015).

22. Wang, S. R. *et al.* Heterozygous mutations in natriuretic peptide receptor-B (NPR2) gene as a cause of short stature. *Hum. Mutat.* **36**, 474–81 (2015).
23. Estrada, K. *et al.* Identifying therapeutic drug targets for rare and common forms of short stature. *bioRxiv* 2020.04.02.022624 (2020) doi:10.1101/2020.04.02.022624.
24. Tajan, M., Paccoud, R., Branka, S., Edouard, T. & Yart, A. The RASopathy Family: Consequences of Germline Activation of the RAS/MAPK Pathway. *Endocr. Rev.* **39**, 676–700 (2018).
25. Tajan, M. *et al.* Noonan syndrome-causing SHP2 mutants impair ERK-dependent chondrocyte differentiation during endochondral bone growth. *Hum. Mol. Genet.* **27**, 2276–2289 (2018).
26. Wu, X. *et al.* MEK-ERK pathway modulation ameliorates disease phenotypes in a mouse model of Noonan syndrome associated with the Raf1L613V mutation. *J. Clin. Invest.* **121**, 1009–1025 (2011).
27. Ono, K. *et al.* The ras-GTPase activity of neurofibromin restrains ERK-dependent FGFR signaling during endochondral bone formation. *Hum. Mol. Genet.* **22**, 3048–62 (2013).
28. Inoue, S.-I., Morozumi, N., Yoshikiyo, K., Maeda, H. & Aoki, Y. C-type natriuretic peptide improves growth retardation in a mouse model of cardio-facio-cutaneous syndrome. *Hum. Mol. Genet.* **28**, 74–83 (2019).
29. Rappold, G. A. *et al.* Deletions of the Homeobox Gene *SHOX* (Short Stature Homeobox) Are an Important Cause of Growth Failure in Children with Short Stature. *J. Clin. Endocrinol. Metab.* **87**, 1402–1406 (2002).
30. Marchini, A., Ogata, T. & Rappold, G. A. A Track record on SHOX: From basic research to complex models and therapy. *Endocrine Reviews* vol. 37 417–448 (2016).
31. Decker, E. *et al.* FGFR3 is a target of the homeobox transcription factor SHOX in limb development. *Hum. Mol. Genet.* **20**, 1524–35 (2011).
32. Nilsson, O. *et al.* Short stature, accelerated bone maturation, and early growth cessation due to heterozygous aggrecan mutations. *J. Clin. Endocrinol. Metab.* **99**, E1510-8 (2014).
33. Gkourogianni, A. *et al.* Clinical Characterization of Patients With Autosomal Dominant Short Stature due to Aggrecan Mutations. *J. Clin. Endocrinol. Metab.* **102**, 460–469 (2017).
34. Domowicz, M. S., Cortes, M., Henry, J. G. & Schwartz, N. B. Aggrecan modulation of growth plate morphogenesis. *Dev. Biol.* **329**, 242–257 (2009).
35. Yasoda, A. *et al.* Overexpression of CNP in chondrocytes rescues achondroplasia through a MAPK-dependent pathway. *Nat. Med.* **10**, 80–86 (2004).
36. Krejci, P. *et al.* Interaction of fibroblast growth factor and C-natriuretic peptide signaling in regulation of chondrocyte proliferation and extracellular matrix homeostasis. *J. Cell Sci.* **118**, 5089–5100 (2005).
37. Moncla, A. *et al.* A cluster of translocation breakpoints in 2q37 is associated with overexpression of NPPC in patients with a similar overgrowth phenotype. *Hum. Mutat.* **28**, 1183–1188 (2007).
38. Ko, J. M. *et al.* Skeletal overgrowth syndrome caused by overexpression of C-type natriuretic peptide in a girl with balanced chromosomal translocation, t(1;2)(q41;q37.1). *Am. J. Med. Genet. Part A* **167**, 1033–1038 (2015).
39. Bocciardi, R. *et al.* Overexpression of the C-type natriuretic peptide (CNP) is associated with overgrowth and bone anomalies in an individual with balanced t(2;7) translocation. *Hum. Mutat.* **28**, 724–731 (2007).
40. Miura, K. *et al.* An Overgrowth Disorder Associated with Excessive Production of cGMP Due to a Gain-of-Function Mutation of the Natriuretic Peptide Receptor 2 Gene. *PLoS One* **7**, e42180 (2012).
41. Miura, K. *et al.* Overgrowth syndrome associated with a gain-of-function mutation of the natriuretic peptide receptor 2 (*NPR2*) gene. *Am. J. Med. Genet. Part A* **164**, 156–163 (2014).

42. Hannema, S. E. *et al.* An Activating Mutation in the Kinase Homology Domain of the Natriuretic Peptide Receptor-2 Causes Extremely Tall Stature Without Skeletal Deformities. *J. Clin. Endocrinol. Metab.* **98**, E1988–E1998 (2013).
43. Kake, T. *et al.* Chronically elevated plasma C-type natriuretic peptide level stimulates skeletal growth in transgenic mice. *Am. J. Physiol. Metab.* **297**, E1339–E1348 (2009).
44. Gonzalez Briceno, L. G. *et al.* Improved general and height-specific quality of life in children with short stature after 1 year on growth hormone. *J. Clin. Endocrinol. Metab.* **104**, 2103–2111 (2019).
45. Center for Disease Control and Prevention. CDC Growth Charts. https://www.cdc.gov/growthcharts/cdc_charts.htm (2000).
46. Richards, S. *et al.* Standards and guidelines for the interpretation of sequence variants: a joint consensus recommendation of the American College of Medical Genetics and Genomics and the Association for Molecular Pathology. *Genet. Med.* **17**, 405–423 (2015).
47. Collett-Solberg, P. F. *et al.* Diagnosis, Genetics, and Therapy of Short Stature in Children: A Growth Hormone Research Society International Perspective. *Horm. Res. Paediatr.* 1–14 (2019) doi:10.1159/000502231.
48. Savarirayan, R. *et al.* Once-daily, subcutaneous vosoritide therapy in children with achondroplasia: a randomised, double-blind, phase 3, placebo-controlled, multicentre trial. *Lancet (London, England)* **396**, 684–692 (2020).
49. Frisancho, A. R. *Anthropometric standards : an interactive nutritional reference of body size and body composition for children and adults*. (University of Michigan Press, 2008).
50. Gerver, W. J. M., Gkourogianni, A., Dauber, A., Nilsson, O. & Wit, J. M. Arm Span and Its Relation to Height in a 2- to 17-Year-Old Reference Population and Heterozygous Carriers of ACAN Variants. *Horm. Res. Paediatr.* **93**, 164–172 (2020).
51. Kelly, A. *et al.* Age-based reference ranges for annual height velocity in US children. *J. Clin. Endocrinol. Metab.* **99**, 2104–2112 (2014).
52. The European QoLISSY Group. *Quality of Life in Short Stature Youth - The QoLISSY Questionnaire User's Manual*. (Pabst Science Publishers, 2013).
